# Identification of p62/SQSTM1 as a component of non-canonical Wnt VANGL2–JNK signalling in breast cancer

**DOI:** 10.1038/ncomms10318

**Published:** 2016-01-12

**Authors:** Tania M. Puvirajesinghe, François Bertucci, Ashish Jain, Pierluigi Scerbo, Edwige Belotti, Stéphane Audebert, Michael Sebbagh, Marc Lopez, Andreas Brech, Pascal Finetti, Emmanuelle Charafe-Jauffret, Max Chaffanet, Rémy Castellano, Audrey Restouin, Sylvie Marchetto, Yves Collette, Anthony Gonçalvès, Ian Macara, Daniel Birnbaum, Laurent Kodjabachian, Terje Johansen, Jean-Paul Borg

**Affiliations:** 1 CRCM, Cell Polarity, Cell signalling and Cancer ‘Equipe labellisée Ligue Contre le Cancer', Inserm, U1068, Marseille F-13009, France; 2 Institut Paoli-Calmettes, Marseille F-13009, France; 3 Aix-Marseille Université, Marseille F-13284, France; 4 CNRS, UMR725, Marseille F-13009, France; 5 CRCM, Molecular Oncology ‘Equipe labellisée Ligue Contre le Cancer', Inserm, U1068, Marseille F-13009, France; 6 Molecular Cancer Research Group, Department of Medical Biology, University of Tromsø—The Arctic University of Norway, Tromsø 9037, Norway; 7 Department of Molecular Cell Biology, Centre for Cancer Biomedicine, University of Oslo and Institute for Cancer Research, The Norwegian Radium Hospital, Oslo N-0310, Norway; 8 Institut de Biologie du Développement de Marseille, Aix-Marseille Université, CNRS UMR 7288, Marseille F-13288, France; 9 CRCM, Marseille Proteomics Platform, Inserm, U1068, Marseille F-13009, France; 10 CRCM, TrGET Platform, Inserm, U1068, Marseille F-13009, France; 11 Department of Microbiology, University of Virginia School of Medicine, Charlottesville, Virginia, Tennessee 37240-7935, USA

## Abstract

The non-canonical Wnt/planar cell polarity (Wnt/PCP) pathway plays a crucial role in embryonic development. Recent work has linked defects of this pathway to breast cancer aggressiveness and proposed Wnt/PCP signalling as a therapeutic target. Here we show that the archetypal Wnt/PCP protein VANGL2 is overexpressed in basal breast cancers, associated with poor prognosis and implicated in tumour growth. We identify the scaffold p62/SQSTM1 protein as a novel VANGL2-binding partner and show its key role in an evolutionarily conserved VANGL2–p62/SQSTM1–JNK pathway. This proliferative signalling cascade is upregulated in breast cancer patients with shorter survival and can be inactivated in patient-derived xenograft cells by inhibition of the JNK pathway or by disruption of the VANGL2–p62/SQSTM1 interaction. VANGL2–JNK signalling is thus a potential target for breast cancer therapy.

## Abstract (web summary)

Defects in non-canonical Wnt/planar cell polarity signalling have recently been linked to breast cancer aggressiveness. Puvirajesinghe *et al*. identify VANGL2, p62/SQSTM1 and JNK as important players in this pathway which may be amenable to therapeutic intervention in breast cancer.

## Graphical abstract

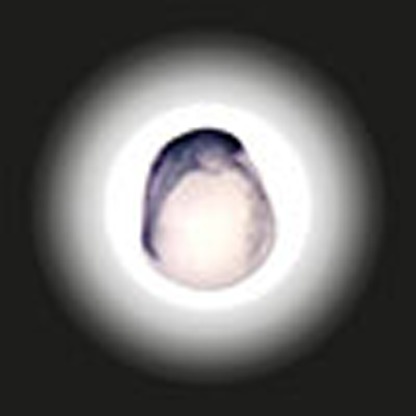

Breast cancer is a molecularly heterogeneous disease that comprises five major subtypes (luminal A and B, ERBB2, basal and normal-like) with different clinical characteristics and prognosis^1^. Basal breast cancer is a very aggressive subtype with high propensity for metastasis formation and poor prognosis^2^. Owing to the lack of hormone receptor (oestrogen receptor (ER) and progesterone receptor (PR)) and ERBB2 expression, patients cannot benefit from hormone therapy or targeted therapy, the only remaining available systemic treatment being standard chemotherapy. Despite new therapeutic approaches such as the optimization of common cytotoxic agents and the testing of novel drugs such as epidermal growth factor receptor (EGFR) and poly-ADP-ribose-polymerase-1 inhibitors, there is still a strong need for novel therapeutic targets for this aggressive breast cancer subtype.

Breast cancer cells commonly reactivate embryonic developmental pathways to promote tumour growth and dissemination. Among these pathways, Wnt signalling plays a crucial role through its involvement in many aspects of the disease, including self-renewal of cancer stem cells, tumour initiation, metastatic development and drug resistance^3^. The Wnt pathway is subdivided into β-catenin-dependent and β-catenin-independent (also called non-canonical) cascades. The latter can be further subdivided into Wnt/calcium and Wnt/planar cell polarity (Wnt/PCP) pathways. The precise mechanism by which Wnt ligands trigger β-catenin-dependent or β-catenin-independent Wnt signalling pathways remains unclear, but probably involves distinct Wnt receptors^3^. Hyperactivation of β-catenin-dependent Wnt signalling has been demonstrated in breast cancer in the late 90s and correlates with poor prognosis^4^,^5^,^6^. Several components of the Wnt/PCP pathway regulate cancer cell motility and invasion, although their involvement in tumorigenesis has long remained elusive. Recent studies have linked upregulation of Wnt/PCP signalling to the development and dissemination of breast cancer^7^ and to poor clinical outcome^8^,^9^. Increased levels of VANGL1–SCRIB and WNT5A/B–FRIZZLED2 correlate with high risk of patient relapse and with progression of late-stage metastatic cancers, respectively. Because targeting this pathway could benefit breast cancer patients^9^, unravelling Wnt/PCP signalling may provide new opportunities for therapeutic intervention.

Wnt/PCP signalling is the least well-characterized Wnt pathway. It regulates biological processes crucial for embryonic development and tissue homeostasis in adults^10^,^11^. The importance of Wnt/PCP genes such as *VANGL2* in developmental processes is best reflected by their involvement in human genetic diseases such as neural tube closure defects^12^. Wnt/PCP signalling, which was initially described in the fruit fly *Drosophila melanogaster*, serves to polarize many epithelial tissues and organizes morphogenetic events in invertebrates and vertebrates^10^,^11^. In addition to *Wnt*, a set of core Wnt/PCP genes in *Drosophila* including *frizzled*, *van gogh* (homologue of human *VANGL1* and *VANGL2*), *scribble*, *prickle*, *dishevelled*, *flamingo*, *fat*, *dachsous* and *diego* constitute a signalling cassette conserved in vertebrates. In invertebrates as well as in vertebrates, the Wnt/PCP pathway leads to activation of small RHO-like GTPases RHOA, RAC1 and c-JUN N-terminal Kinase (JNK)^3^. The underlying mechanism by which Wnt/PCP signalling activates JNK remains unclear. In addition to its role in morphogenesis, JNK is involved in apoptosis, cell proliferation and cell motility, and can contribute to tumour promotion or inhibition depending on the cellular and tissue context^13^.

Here we report the overexpression of the Wnt/PCP core component VANGL2 in breast cancers with poor prognosis. We demonstrate the involvement of VANGL2 in tumour growth in cell culture and mice. We identify p62/sequestosome-1 (hereafter named p62/SQSTM1) as a novel VANGL2-binding partner in breast cancer cells. p62/SQSTM1 is an intracellular Phox and Bem1p (PB1) domain-containing scaffold protein involved in important processes such as selective autophagy, cell signalling and induction of epithelial–mesenchymal transition (EMT)^14^,^15^. p62/SQSTM1 has been linked to several diseases such as Paget's disease of bone, neurodegenerative diseases, liver disorders and cancer^16^. It is overexpressed in breast cancers, including aggressive basal and ERBB2 subtypes, and involved in many aspects of oncogenesis^17^,^18^,^19^,^20^. We find that p62/SQSTM1 is required to recruit and activate JNK in breast cancer cells through an evolutionarily conserved VANGL2–p62/SQSTM1–JNK signalling cascade. This proliferative pathway is upregulated in breast cancer patients with shorter survival and in patient-derived xenografts (PDXs), and is sensitive to inhibition of JNK and of the VANGL2–p62/SQSTM1 interaction. These data describe a non-canonical Wnt/PCP pathway overexpressing VANGL2 in aggressive breast cancer and identify p62/SQSTM1 as an important player in VANGL2–JNK signalling.

## Results

### Overexpression of *VANGL2* in breast cancer

**Figure 1:**
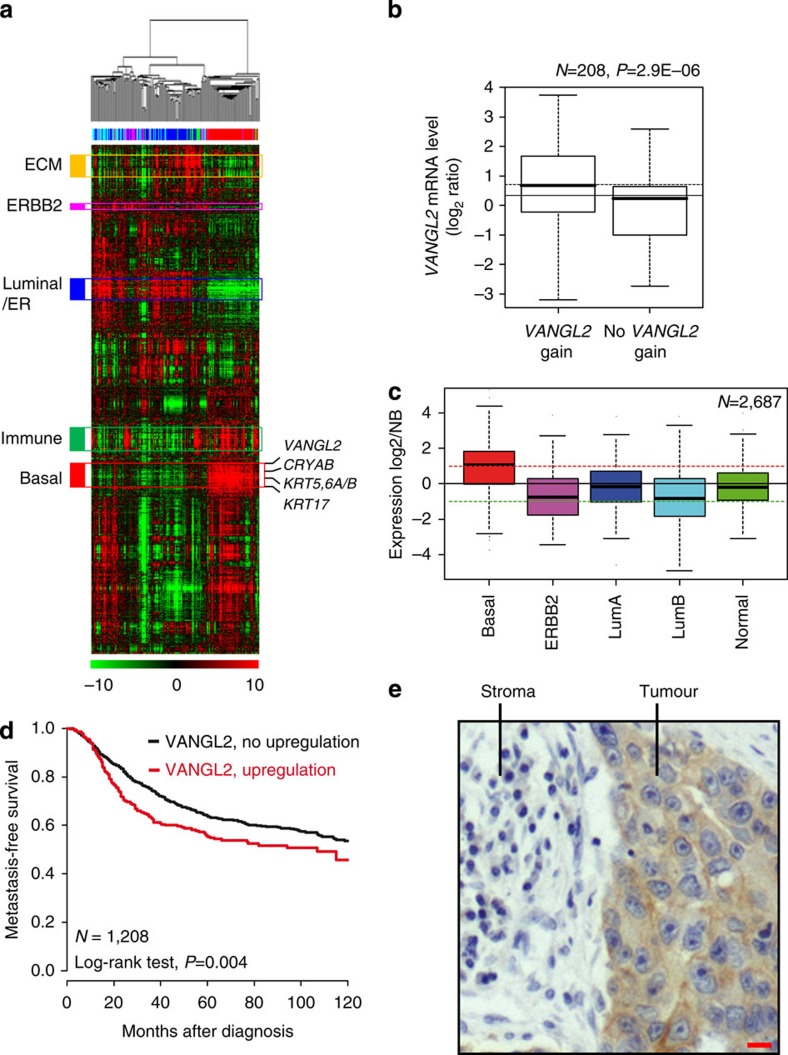
Overexpression of *VANGL2* in breast cancer. (**a**) Hierarchical clustering of the 208 breast cancers and 4 NB samples (columns) and the 12,304 most variable genes (rows). According to a log_2_ pseudocolour scale (bottom), red indicates a high level of mRNA expression compared with the median value across all samples, whereas green indicates a low level of expression. The magnitude of deviation from the median is represented by the colour saturation. The dendrogram of samples (above matrices) represents overall similarities in gene expression profiles. To the left of the colour matrix are represented some biologically relevant gene clusters (orange: extracellular matrix cluster (ECM); pink: ERBB2 cluster; blue: luminal/ER cluster; green: immune cluster; red: basal cluster). A few genes of the basal cluster are shown, including *VANGL2*, as well as classical basal genes (*KRT5*, *KRT6*, *KRT17* and *CRYAB*). (**b**) Box and whisker plots of *VANGL2* expression across 208 breast cancer samples profiled by both expression DNA arrays and aCGH, and according to (Student's *t*-test) *VANGL2* genomic status: with (left, 104 samples) versus without (right, 104 samples) gain defined as a DNA copy number ratio tumour/NB⩾1.5). (**c**) Box and whisker plots of *VANGL2* expression across 2,687 breast cancer samples according to molecular subtypes. Expression values are NB-centred. The horizontal black line represents the level of expression of *VANGL2* in NB tissue. Differences between the subtypes were tested for significance using one-way analysis of variance (ANOVA). For each box and whisker plot, the median value and interquartile ranges are indicated. (**d**) Kaplan–Meier MFS curves in breast cancer patients according to *VANGL2* mRNA expression. The 5-year MFS are 55% (upregulation; *N*=296) and 64% (absence of upregulation; *N*=912). (**e**) Immunohistochemistry experiment using anti-VANGL2 2G4 monoclonal antibody (mAb) shows that VANGL2 is more expressed in tumour cells (tumour) than in the stromal tissue (stroma) in basal breast cancer. Scale bar, 10 μM.

To address the role of VANGL2 in breast cancer, we first examined *VANGL2* messenger RNA expression in a large data set of 2,687 invasive breast cancers (Supplementary Table 1). Whole-genome clustering showed that *VANGL2* was part of the ‘basal' gene cluster that also includes *KRT5/6/17* and *CRYAB* genes (Fig. 1a). A total of 625 tumours (23%) showed *VANGL2* mRNA upregulation compared with normal breast (NB). The analysis of available array-comparative genomic hybridization (aCGH) data from 208 samples revealed that half of them show a gain of *VANGL2* DNA copy number, which is statistically correlated with mRNA upregulation (Student's *t*-test, *P*<0.001; Fig. 1b). Histoclinical correlations (Supplementary Table 2) revealed that *VANGL2* upregulation is associated with larger tumour size (Fisher's exact test, *P*=0.009), ER-negative (Fisher's exact test, *P*<0.001), PR-negative (Fisher's exact test, *P*<0.001) and ERBB2-negative (Fisher's exact test, *P*=0.017) immunohistochemistry (IHC) status, triple-negative status (Fisher's exact test, *P*<0.001) and basal subtype (Fisher's exact test, *P*<0.001; Fig. 1c), and tends to be associated with higher grade (Fisher's exact test, *P*=0.067). *VANGL2* upregulation correlates with poor metastasis-free survival (MFS) in both univariate (log-rank test, *P*=0.004; Fig. 1d) and multivariate (Wald test, *P*=0.005) analyses (Supplementary Table 3). IHC analysis for VANGL2 using 2G4 monoclonal antibody^21^ showed that the VANGL2 protein is abundant in epithelial cancer cells of patient tumours compared with surrounding tissues, suggesting a cell-autonomous activity in tumours (Fig. 1e). Epithelial expression of *VANGL2* was confirmed in a panel of 35 breast cancer cell lines previously profiled using DNA microarrays^22^. *VANGL2* expression was found to be heterogeneous across cell lines and higher in the basal and mesenchymal cell lines, such as SUM149 and SKBR7, than in the luminal ones (Supplementary Fig. 1), as observed in the clinical cancer samples.

### VANGL2 is implicated in tumour growth

**Figure 2:**
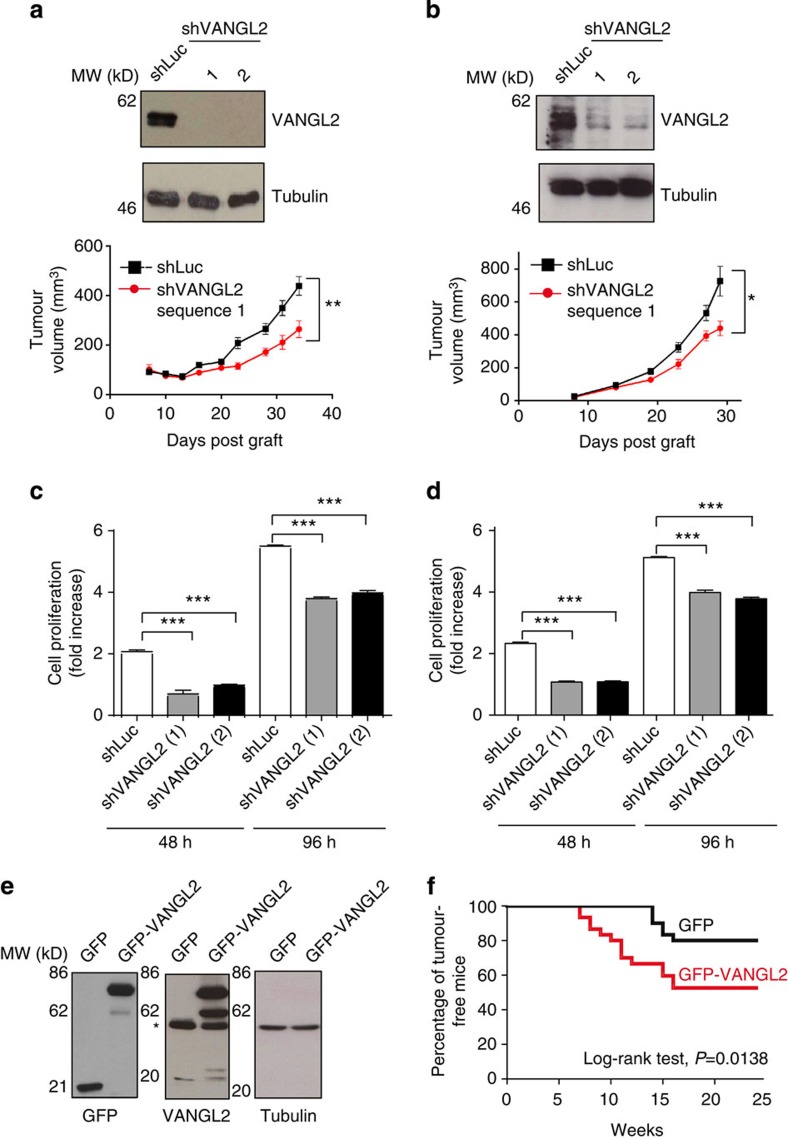
VANGL2 participates in tumour growth in cell culture and murine experiments. (**a**) Basal cell lines chosen for their high basal score/correlation were SUM149 *r*=0.36 (**a**) and HCC1806 (**b**) *r*=0.28 and threshold was 0.15. Expression of two short hairpin RNAs abrogated VANGL2 expression in SUM149 cells by western blot analysis (upper panels). SUM149 cells (5 × 10^6^) were subcutaneously inoculated into the right flank of 4–6-week-old female NSG mice. Tumoral volume was measured at different times (lower panels). The mean and s.e.m. values (*n*=6, for shLuc and shVANGL2-transfected cells). The statistical significance between the data sets was determined using a two-way ANOVA test. *P≤0.05, **P≤0.005. (**b**) Same as **a** using HCC1806 cells, except that 1 × 10^6^ cells were inoculated into NSG mice. (**c**,**d**) Downregulation of VANGL2 with two different shRNAs led to a decreased proliferation of SUM149 (**c**) and HCC1806 (**d**) cells. Error bars represent mean±s.d. (**e**) COMMA-D cells were transduced with lentiviral supernatants allowing expression of GFP or GFP–VANGL2. Cell extracts were probed by western blot analysis with anti-GFP, -VANGL2 and -tubulin antibodies. An asterisk pinpoints endogenous VANGL2. (**f**) Kaplan–Meier curve of tumour-free status of mice transplanted with COMMA-D cells overexpressing GFP or GFP–VANGL2 (*n*=30). The statistical significance between the data sets was determined using a log-rank test.

To assess the functional importance of VANGL2 in basal breast cancer cells, VANGL2 protein levels were stably reduced using two short hairpin RNAs (shVANGL2 seq1 and seq2) in SUM149 (Fig. 2a) and HCC1806 (Fig. 2b) cells. In both cases, VANGL2 was efficiently knocked down (>90%; Fig. 2a,b, upper panels). VANGL2-depleted SUM149 (Fig. 2a, lower panel, and Supplementary Fig. 2) and HCC1806 (Fig. 2b, lower panel) cells xenografted into NOD/SCID/γc null female mice (NSG) showed reduced *in vivo* tumour growth compared with cells transfected with shLuc. The reduced tumour growth of VANGL2-depleted cells was confirmed by *in vitro* assays. First, as previously shown in other cell models^23^, we found that VANGL2 depletion impaired SUM149-directed cell migration in Boyden chamber assays (Supplementary Fig. 2). Second, we tested anchorage-dependent and -independent proliferation and observed that loss of VANGL2 decreased the proliferation rates of SUM149 (Fig. 2c and Supplementary Fig. 2) and HCC1806 cells (Fig. 2d). To determine whether overexpression of VANGL2 participates in tumorigenesis, green fluorescent protein (GFP)-tagged VANGL2 or GFP alone were transduced and stably expressed in a preneoplastic mouse mammary epithelial *cell* line, COMMA-D. Respective protein expression levels were monitored (Fig. 2e) and the cells were injected into the pre-cleared fat pad of syngeneic female BALB/c mice as previously described^24^. In this mouse model of breast cancer, we observed that overexpressed VANGL2 caused a dramatic reduction in tumour latency, since tumour occurrence was detectable 7 weeks post graft with VANGL2-overexpressed cells, whereas 14 weeks were required for GFP-expressed control cells (Fig. 2f). Overall, these data suggest that VANGL2 overexpression participates in tumour growth in breast cancer cell lines.

### VANGL2 binds to p62/SQSTM1

**Figure 3:**
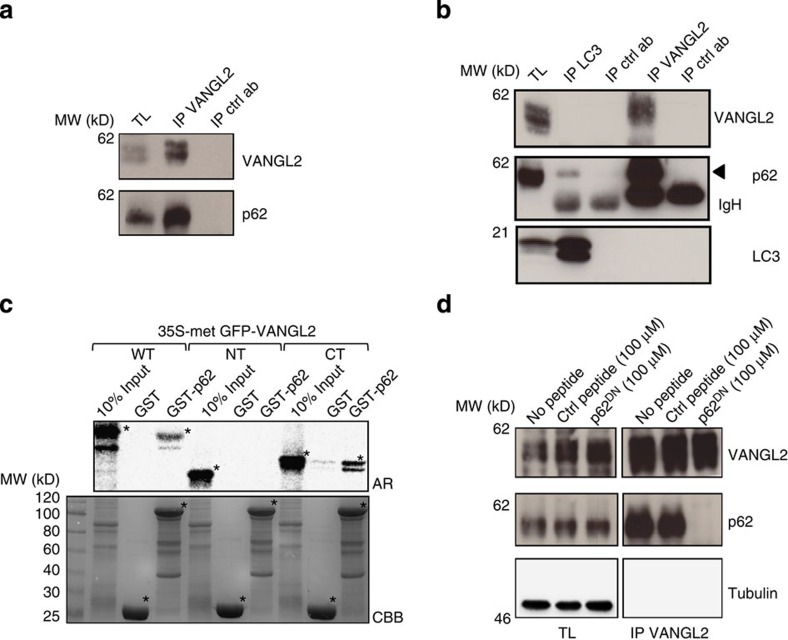
Identification of the signalling adapter p62/SQSTM1 as a direct binding partner of VANGL2. (**a**) Endogenous interaction between VANGL2 and p62/SQSTM1 is revealed by western blot analysis after immunoprecipitation using SKBR7 cell extracts. TL is total lysate. Crtl ab is an isotype-matched antibody. (**b**) The VANGL2–p62/SQSTM1 interaction occurs independently of LC3. Proteins were extracted from SUM149 cells and co-immunoprecipitations were carried out with the indicated antibodies. LC3 co-precipitates with p62/SQSTM1 but not with VANGL2. Reciprocally, VANGL2 co-immunoprecipitates with p62/SQSTM1 but not with LC3. IP control antibodies (IP crtl ab) are a polyclonal rabbit (for IP LC3) and a monoclonal rat antibody (for IP VANGL2). IgHs are immunoglobulin heavy chains. (**c**) GST pulldown assays of *in vitro* translated GFP–VANGL2 (full length: WT, N-terminal 1–102: NT, C-terminal 242–521: CT) showed that VANGL2 WT and CT directly bind to GST–p62/SQSTM1 (GST-p62) but not to GST. Asterisks indicate *in vitro* translated VANGL2 (top panel) and GST (bottom panel) proteins. AR, autoradiography; CBB, Coomassie Brilliant Blue. (**d**) A p62/SQSTM1 peptide (p62^DN^) disrupts the endogenous VANGL2–p62/SQSTM1 complex. SUM149 cell protein extracts were incubated with the indicated peptides p62^DN^ or scrambled control peptide (Ctrl peptide) at 100 μM. VANGL2 was then immunoprecipitated (IP VANGL2) and bound proteins were immunoblotted with the indicated antibodies. TLs showed that equal amounts of proteins were present in each condition.

To identify the molecular mechanisms underlying the regulation of tumorigenesis by VANGL2, endogenous VANGL2 protein complexes were immunoprecipitated from SUM149 cells and identified using liquid chromatography and mass spectrometry analysis. In addition to VANGL1, a known VANGL2 partner^21^, a cytoplasmic PB1 domain-containing protein, p62/SQSTM1, was identified (Table 1 and Supplementary Data 1) in the anti-VANGL2 immunoprecipitate but not in the isotype-matched antibody control experiment. We validated the mass spectrometry results with western blot analysis using anti-p62/SQSTM1 antibody as a probe following immunoprecipitation of VANGL2 in SUM149 (Fig. 3a) and SKBR7 (Supplementary Fig. 3) cells. Whereas immunoprecipitated p62/SQSTM1 co-purified efficiently with VANGL2 (Supplementary Fig. 3), the p62/SQSTM1 partner LC3B/ATG8 (ref. 25) was not detected in the VANGL2-associated complex (Fig. 3b), revealing the likely existence of different pools of p62/SQSTM1. We have thus identified a previously uncharacterized VANGL2–p62/SQSTM1 complex in breast cancer cells.

### Characterization of the VANGL2–p62/SQSTM1 interaction

To determine whether VANGL2 is able to directly bind to its endogenous partners, *in vitro* translated GFP–VANGL2 was produced in a cell-free system and used in pulldown assays. Glutathione *S*-transferase (GST)–p62/SQSTM1 (GST–p62) was able to bind to GFP–VANGL2, whereas no binding was obvious with the GST control protein (Fig. 3c). VANGL2 is a transmembrane protein presenting two cytoplasmic regions, at the N terminus (1–102) and C terminus (242–521) (Supplementary Fig. 3). The (242–472) sequence of VANGL2 was found to be responsible for the p62/SQSTM1 interaction (Fig. 3c and Supplementary Fig. 3), which is independent of the Dishevelled- and Scribble-binding regions as shown by mutagenesis of the VANGL2 carboxyl-terminal tail (Supplementary Fig. 3)^23^,^26^.

p62/SQSTM1 is a selective autophagy receptor and a signalling scaffold protein harbouring PB1, Zn (zinc finger), TB (TRAF6 binding), LIR (LC3-interacting region), KIR (KEAP1-interacting region) and UBA (ubiquitin-associated) domains (Supplementary Fig. 3)^14^. To characterize the p62/SQSTM1 region required for VANGL2 binding, a screen was carried out using a panel of p62/SQSTM1 mutants. We found that PB1-dependent self-association of p62/SQSTM1 (ref. 27) was dispensable for the integrity of the complex, whereas a sequence lying carboxyl-terminal to the LIR/KIR domains was necessary for the interaction (Supplementary Fig. 3). We generated a synthetic p62/SQSTM1 peptide mimicking the interaction sequence thus identified, p62^346-388^ (hereafter named p62^DN^), and tested its ability to inhibit the endogenous VANGL2–p62/SQSTM1 interaction in comparison with a scrambled control peptide. A complete inhibition was observed with p62^DN^ but not with the control peptide used at concentration of 100 μM (Fig. 3d). The p62^DN^ peptide exerted dose-dependent inhibition. Indeed, incubation of low amounts of p62^DN^ (5 μM) was sufficient to partially inhibit the VANGL2–p62/SQSTM1 interaction (Supplementary Fig. 3). Further analysis with mass spectrometry showed that p62^DN^ treatment at 50 μM led to the loss of interaction between p62/SQSTM1 and VANGL2, thus confirming the western blot results (Supplementary Fig. 3 and Supplementary Data 2). Interestingly, this experiment also showed that the VANGL1–VANGL2 interaction, that occurs through heterodimerization^21^, is unaffected by peptide inhibition, demonstrating that VANGL2 interaction with p62/SQSTM1 is independent of its interaction with VANGL1 (Supplementary Fig. 3). Overall, we identify p62/SQSTM1 as a direct VANGL2 partner and describe a p62/SQSTM1 motif required for the interaction.

### Localization of the VANGL2–p62/SQSTM1 complex

**Figure 4:**
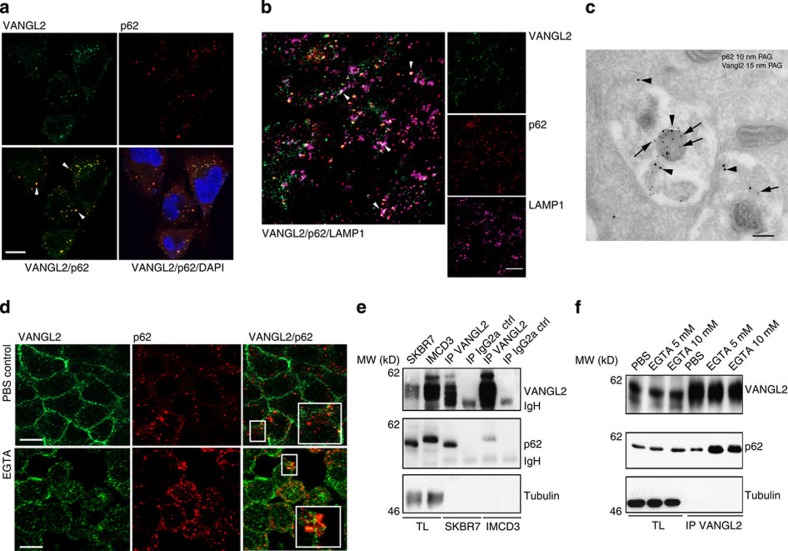
Colocalization of VANGL2 and p62/SQSTM1 in late endosomes. (**a**) Immunofluorescence staining of SKBR7 cells showed colocalization of endogenous VANGL2 (green) and p62/SQSTM1 (red) in discrete cytoplasmic puncta. Scale bar, 10 μm. The mean Pearson correlation for VANGL2 and p62/SQSTM1 is 0.62, calculated using the Image J software for ∼15 cells per field of view and from 10 images. (**b**) Partial colocalization of VANGL2 and p62/SQSTM1 in late endosomes of SKBR7 cells stained with the LAMP1 marker. Scale bar, 20 μm. (**c**) SKBR7 ells were cultured in a nutrient-deprived medium and treated with 100 nM bafilomycin A1 (6 h) before fixation. Double labelling against VANGL2 (arrowheads) and p62/SQSTM1 (arrows), as described in Methods, showed accumulations of both markers in vesicular structures probably resembling endosomes/amphisomes. Scale bar, 200 nm. (**d**) IMCD3 cells were treated with PBS or EGTA (5 mM) for 30 min. Immunofluorescence and confocal analysis were performed using the indicated antibodies. Scale bar, 10 μm. Inserts show colocalized VANGL2 and p62/SQSTM1. (**e**) The VANGL2–p62/SQSTM1 complex was recovered in confluent SKBR7 or IMCD3 cells with 2G4 mAb (IP VANGL2) but not a control antibody (IP IgG2a ctrl) as seen using western blot analysis with the indicated antibodies. The complex was more abundant in cancer cells (SKBR7) than in polarized cells (IMCD3). Note that human (SKBR7 cells) and murine (IMCD3 cells) p62/SQSTM1 run at different molecular weights. (**f**) Lysates of confluent IMCD3 cells treated for 30 min with PBS or EGTA were subjected to 2G4 mAb immunoprecipitation (IP VANGL2). Immunoprecipitated proteins were probed by western blot analysis with the indicated antibodies. Increased amounts of VANGL2–p62/SQSTM1 complexes were recovered after EGTA treatment.

To identify the subcellular localization of the VANGL2–p62/SQSTM1 complex in breast cancer cells, SKBR7 cells were analysed by immunofluorescence and by confocal microscopy. VANGL2 was found to display a predominately punctate cytoplasmic pattern in the perinuclear region, as reported in precedent studies^28^, and to colocalize with p62/SQSTM1 (Fig. 4a). p62/SQSTM1 was previously detected in late endosomal compartments^29^. To define whether VANGL2 and p62/SQSTM1 colocalize in these compartments, a three-colour immunofluorescence and confocal analysis was performed with LAMP1, a late endosomal/lysosomal compartment marker. We found a substantial colocalization between LAMP1, VANGL2 and p62/SQSTM1 in breast cancer cells (Fig. 4b). The colocalization between VANGL2 and p62/SQSTM1 was further confirmed by immunoelectron microscopy, which showed accumulation of both markers in vesicular structures resembling endosomes/amphisomes (Fig. 4c and Supplementary Fig. 4). Because VANGL2 has also been described as a plasma membrane protein in non-tumoral polarized epithelial cells^21^, we studied VANGL2–p62/SQSTM1 colocalization and interaction in polarized IMCD3 cells. In these cells, as expected, VANGL2 is strongly recruited to the plasma membrane and nicely colocalizes with β-catenin at cell–cell contacts (Supplementary Fig. 4). It shows weak colocalization with p62/SQSTM1 in intracellular vesicles (Fig. 4d, upper panels). In contrast, SKBR7 cells poorly polarize, as shown by the irregular β-catenin staining, and have barely detectable plasma membrane VANGL2 (Supplementary Fig. 4). Co-immunoprecipitation between VANGL2 and p62/SQSTM1 was more efficient in SKBR7 than in IMCD3 cells despite a stronger expression of VANGL2 in IMCD3 cells and comparable amounts of p62/SQSTM1 in both cell types (Fig. 4e). When the cell junctions of polarized IMCD3 cells were disrupted by adding a calcium chelation agent (EGTA), VANGL2 relocalized from the plasma membrane to intracellular vesicles, and its colocalization (Fig. 4d, lower panels) and co-immunoprecipitation with p62/SQSTM1 (Fig. 4f) increased. Altogether, these data suggest that the VANGL2–p62/SQSTM1 colocalization occurs in part in late endosomal compartments in breast cancer cells and is regulated by cell junction formation.

Because p62/SQSTM1 plays a key role in the autophagic degradation of polyubiquitinylated proteins^14^, it was important to establish whether VANGL2 is degraded by autophagy. Inhibition of autophagy by serum starvation and mammalian target of rapamycin (mTOR) inhibition (rapamycin treatment) led to the expected cleavage of LC3, whereas VANGL2 levels remained unchanged in western blot experiments (Supplementary Fig. 4). Moreover, no change in the VANGL2 expression level was observed in mouse embryonic fibroblasts lacking p62/SQSTM1 expression on autophagy induction or inhibition (Supplementary Fig. 4). These results showed that VANGL2 is resistant to autophagic degradation. In contrast, we found that VANGL2 was sensitive to a proteasome degradation pathway (Supplementary Fig. 4), as was previously described for its paralogue VANGL1 (ref. 30).

### p62 recruits JNK to VANGL2 and contributes to its activation

**Figure 5:**
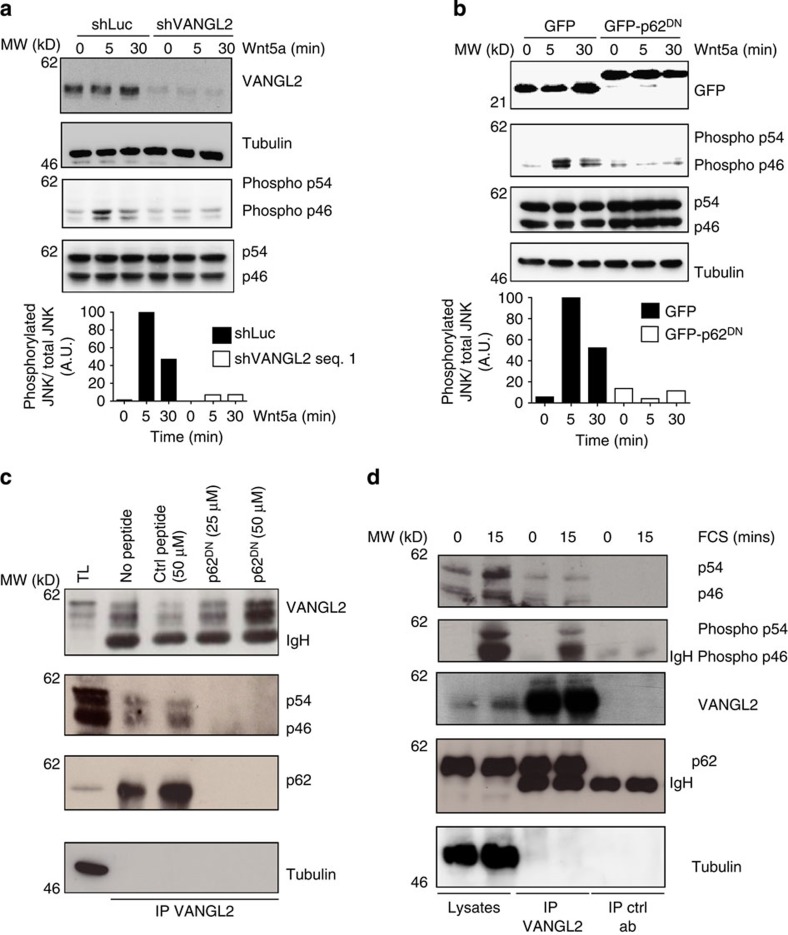
The VANGL2–p62/SQSTM1 complex regulates JNK phosphorylation. (**a**) Downregulation of VANGL2 in SUM149 cells using a specific shRNA led to reduced JNK phosphorylation induced by Wnt5a (100 ng ml^−1^ for the indicated times). JNK is represented by two isoforms: p54 and p46. Wnt5a led to p46 (phospho-p46) and not p54 (phospho-54) phosphorylation. Relative quantification of immunoblots (phosphorylated JNK/total JNK) is representative from three independent experiments and use of two different shRNAs. (**b**) Expression of GFP-p62^DN^, but not GFP, in SUM149 cells led to decreased p46 JNK phosphorylation (phospho-46) induced by 100 ng ml^−1^ of Wnt5a at the indicated times. Relative quantification of immunoblots (phosphorylated JNK/total JNK) is representative from three independent experiments. (**c**) SUM149 cell extracts were added with the control peptide (Ctrl peptide) or the p62/SQSTM1 peptide (p62^DN^) that inhibited recruitment of JNK and p62/SQSTM1 to VANGL2. (**d**) Proteins extracted from SUM149 cells treated or not with serum were immunoprecipitated with anti-VANGL2 antibody and blotted with the indicated antibodies. p62/SQSTM1, JNK and phosphorylated JNK (phospho-p54 and phospho-p46) were present in the VANGL2 complex.

To determine the role of p62/SQSTM1 in VANGL2 signalling, we assessed its impact on the regulation of JNK activation, a well-known downstream effector of VANGL2 (ref. 26), represented by two isoforms p54 and p46. For this purpose, we first confirmed that stimulation of SUM149 cells with WNT5A, a Wnt/PCP ligand, leads to JNK activation as monitored by the phosphorylation state of p46 JNK, in a VANGL2-dependent manner. As illustrated in Fig. 5a, on WNT5A stimulation, depletion of VANGL2 by a specific short hairpin RNA (shRNA) led to a significant reduction in p46 JNK phosphorylation compared with shLuc control cells. As observed for VANGL2, p62/SQSTM1 depletion by two different short interfering RNAs (siRNAs) led to a two- to threefold decrease in JNK phosphorylation in response to WNT5A treatment, suggesting that JNK activation by this WNT ligand is dependent on VANGL2 and p62/SQSTM1 (Supplementary Fig. 5).

We assessed whether the VANGL2–p62/SQSTM1 interaction contributes to JNK phosphorylation using GFP-p62^DN^, a fusion protein containing the inhibitory p62^DN^ peptide (Fig. 3e), which is able to inhibit the VANGL2–p62/SQSTM1 interaction as revealed by co-immunoprecipitation experiments (Supplementary Fig. 5). Overexpression of GFP-p62^DN^ impaired JNK phosphorylation following WNT5A stimulation (Fig. 5b), suggesting that JNK activation by WNT5A requires integrity of the VANGL2–p62/SQSTM1 complex. This result led us to investigate a possible molecular link between JNK and the VANGL2–p62/SQSTM1 complex. We thus carried out immunoprecipitations of VANGL2 in the presence of p62^DN^ or control soluble synthetic peptides and looked for JNK interaction. As illustrated in Fig. 5c, JNK was detectable in VANGL2 immunoprecipitates only when the VANGL2–p62/SQSTM1 interaction was preserved. This result was confirmed by immunoprecipitation of proteins extracted from SUM149 cells treated by a p62/SQSTM1 siRNA. Under such conditions, JNK was unable to interact with VANGL2 (Supplementary Fig. 5). Finally, we addressed whether the interaction between JNK and the VANGL2–p62/SQSTM1 complex was modulated by activation in co-immunoprecipitation experiments (Fig. 5d and Supplementary Fig. 5). We observed that serum or Wnt5a stimulation did not affect VANGL2–p62/SQSTM1 complex formation or its interaction with JNK. JNK was found to be phosphorylated on stimulation and remained associated with the VANGL2–p62/SQSTM1 complex. Altogether, we provide compelling evidence that, in breast cancer cells, WNT5A–VANGL2 signalling requires the integrity of the VANGL2–p62/SQSTM1 complex to associate with and phosphorylate JNK.

### p62/SQSTM1 is involved in a conserved WNT5A–VANGL2–JNK signalling pathway

**Figure 6:**
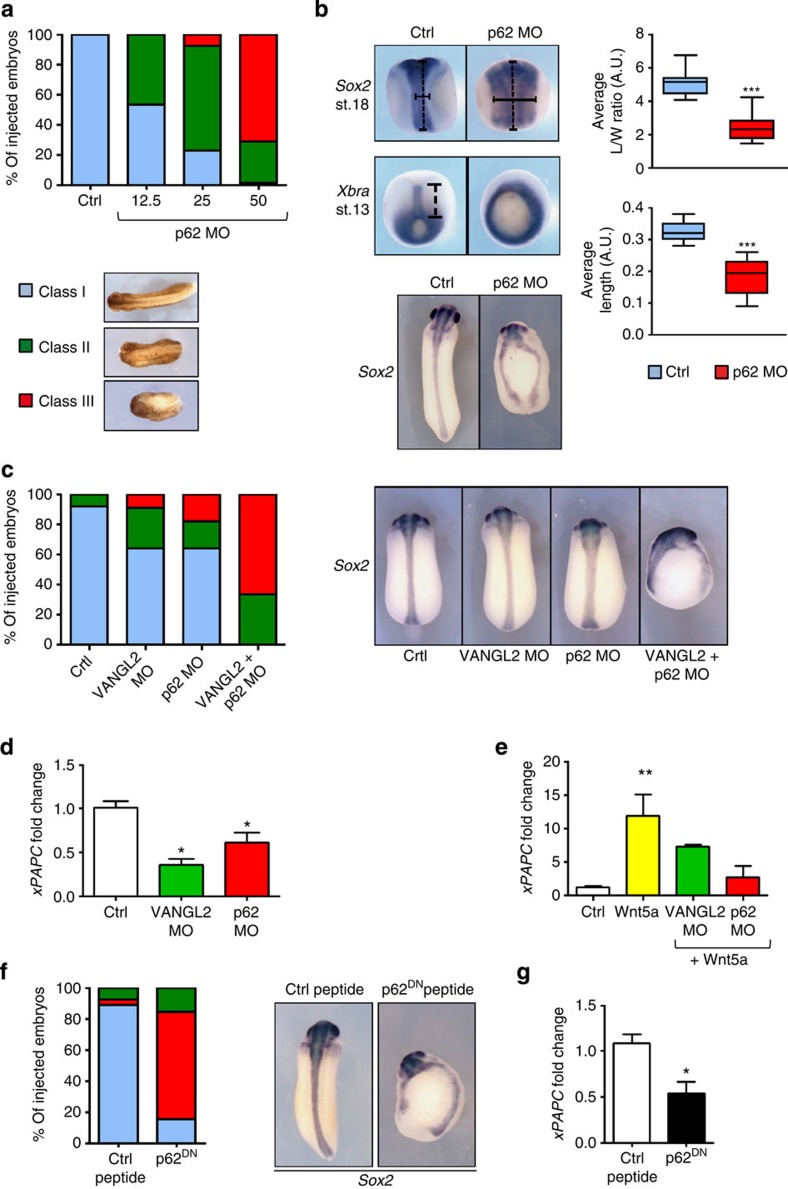
p62/SQSTM1 is necessary for VANGL2 pathway-mediated *in vivo* morphogenesis. (**a**) Two-cell embryos were injected in each blastomere with 12.5 (*n*=15), 25 (*n*=13) and 50 (*n*=104) ng of p62 MO. Morphology was analysed at tailbud stage. (**b**) Embryos injected with 50 ng of p62 MO were processed for WISH analysis at mid-neurula and late gastrula stages. Convergence extension in the neural tube of late neurula embryos was evaluated by the average length/width ratio of the *Sox2* domain (*n*=17). Convergence extension in the axial mesoderm of late gastrula embryos was evaluated by the average length of the *Xbra* domain (*n*=16). Tailbud embryos were stained with the *Sox2* probe to highlight neural tube defect (bottom panel, *n*=30 control, *n*=104 morphants). (**c**) Two-cell embryos were injected in each blastomere with 11.5 ng of VANGL2 MO (*n*=27), 8 ng of p62 MO (*n*=26) or 11.5 ng of VANGL2+8 ng of p62 MO (*n*=32). Morphology was analysed at the tailbud stage using criteria of **a**. These embryos were processed for analysis of *Sox2* expression at the tailbud stage (*n*=3). (**d**) Ten embryos injected as in **b** or with 34.5 ng of VANGL2 MO in each blastomere were collected at stage 13 and processed for RT–qPCR. (**e**) Four-cell embryos injected with Wnt5a mRNA (30 pg per cell) in the animal pole received a second injection of VANGL2 (11.5 ng per cell), or p62 (12.5 ng per cell) in all animal blastomeres at eight-cell stage. Fifteen animal caps per condition were isolated at the blastula stage, cultured for 4 h (at 23 °C) and then processed for RT–qPCR. (**f**) Two-cell embryos were injected in each blastomere with 4.5 ng of control peptide (*n*=27) or p62^DN^ peptide (*n*=26). Morphology was analysed as in **a** and processed for analysis of *Sox2* expression at the tailbud stage. (**g**) Ten embryos injected as in **f** were collected at stage 13 and processed for RT–qPCR. For qPCR graphs, error bars represent s.e.m. values of three or more independent experiments with two technical duplicates. Statistical analyses used unpaired Student's *t*-test, except in **e** where one-way ANOVA with Dunnett's test (99.9% confidence intervals) were applied. *P≤0.05; **P≤0.005; ***P≤0.0005; Ctrl, control; MO, morpholino; mRNA, messenger RNA; RT–qPCR, reverse transcriptase–quantitative PCR; WISH, whole-mount *in situ* hybridization.

To determine whether the VANGL2–p62/SQSTM1 complex and its involvement in JNK signalling is uniquely linked to pathological conditions, or whether it also has a physiological relevance, we tested its involvement in *Xenopus* embryogenesis^26^,^31^. First, we confirmed that *p62/SQSTM1* is conserved in *Xenopus* (Supplementary Fig. 6). We found that the expression pattern of *p62/SQSTM1* overlaps with that of *VANGL2* (ref. 31) as both genes are rather ubiquitously expressed during gastrulation and neurulation (Supplementary Fig. 6). Inhibition of the function of *p62/SQSTM1* by injection of a morpholino oligonucleotide (MO) led to VANGL2-like dose-dependent phenotypes^31^ including incomplete neural tube closure and severely reduced axial elongation (Fig. 6a,b). As typically observed when Wnt/PCP activity is perturbed, mesoderm and neural tissue cells were normally specified in p62/SQSTM1 morphant embryos, but their capacity to undergo convergence extension was severely impaired (Fig. 6b). Embryos developed normally when injected with a *p62/SQSTM1* MO carrying five mismatches (Supplementary Fig. 6). In contrast, a second independent MO targeting *p62/SQSTM1* 5′ untranslated repeat also caused neural tube defects, albeit at lower frequency (Supplementary Fig. 6). To check whether the most active *p62/SQSTM1* MO induced a specific phenotype, we performed a rescue assay with a construct encoding the human p62/SQSTM1 protein (Supplementary Fig. 6). We observed that expression of human p62/SQSTM1 could efficiently reduce morphological anomalies caused by *Xenopus p62/SQSTM1* knockdown in tailbud embryos, and correct blastopore closure, convergent extension and neural tube closure. The observed rescue further confirmed the functional conservation of p62/SQSTM1 proteins between *Xenopus* and human. The anticipated cooperation between *VANGL2* and *p62/SQSTM1* was demonstrated by concomitant knockdown, using suboptimal amounts of the respective MOs, which yielded stronger phenotypes than separate injections (Fig. 6c).

We next tested whether VANGL2 and its partner p62/SQSTM1 are required for JNK-mediated gene expression in *Xenopus*. Expression of a Wnt/PCP effector gene *Xenopus* Paraxial Protocadherin (*xPAPC*)^32^, which is controlled by a WNT5A/JNK pathway^33^, was downregulated by knockdown of *VANGL2* and *p62/SQSTM1* in developing embryos, or following *Wnt5A* mRNA injection in animal caps (Fig. 6d,e). The physiological importance of the VANGL2–p62/SQSTM1 interaction was assessed by injection of the competing human p62^DN^ peptide (Fig. 3e), which has extensive similarities with the corresponding *Xenopus* sequence. Treatment with this peptide, but not with the scrambled peptide, caused severe neural tube closure defects and reduced *xPAPC* expression, suggesting that JNK signalling was impaired (Fig. 6f,g). From these experiments, we conclude that p62/SQSTM1 expression, as well as interaction with VANGL2, contribute to VANGL2-dependent functions in *Xenopus*. We have thus assigned p62/SQSTM1 to a conserved WNT5A–VANGL2–JNK signalling pathway required for proper morphogenesis of the *Xenopus* embryo.

### VANGL2–p62/SQSTM1–JNK signalling in breast cancer

**Figure 7:**
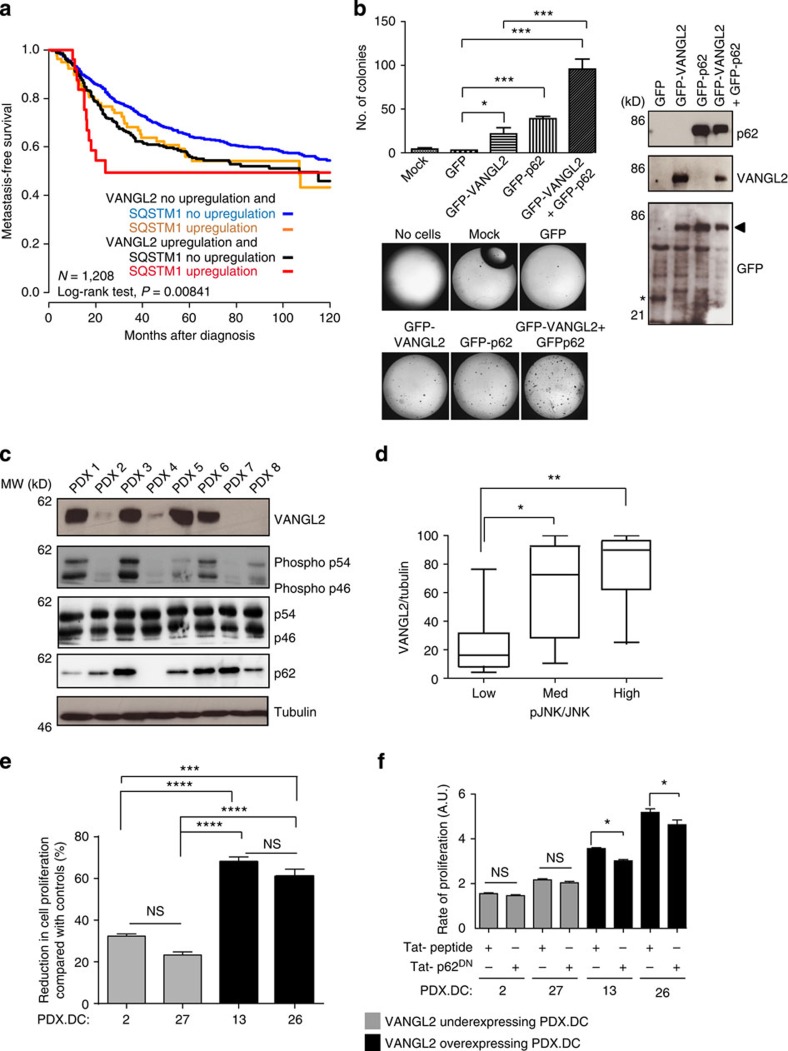
Disruption of the VANGL2–p62/SQSTM1 interaction in breast cancer cells. (**a**) Kaplan–Meier MFS curves of breast cancer patients according to concomitant *VANGL2* and *p62/SQSTM1* mRNA expression. The 5-year MFS are 49% (both upregulated; *N*=27), 64% (both not upregulated; *N*=833) and 56% (one upregulated, the other not upregulated; *N*=79 and *N*=269). (**b**) Soft agar colony formation of T47D cells overexpressing GFP, GFP–VANGL2 and GFP–p62/SQSTM1 (right). Protein expression was revealed with anti-p62/SQSTM1, anti-VANGL2 and anti-GFP antibodies by western blot analysis (right). In anti-GFP blot, the arrowhead indicates position of co-migrating GFP–VANGL2 and GFP–p62/SQSTM1 and the asterisk pinpoints GFP alone. Error bars represent mean±s.d. (*n*=3). (**c**) Protein levels of VANGL2, phosphorylated JNK, JNK (p54/p46), p62/SQSTM1 and tubulin assessed in eight breast cancer PDXs (PDX 1–8) by western blot analysis. (**d**) VANGL2, JNK, phosphorylated JNK and tubulin signals from 30 PDX protein extracts were quantified. VANGL2/tubulin ratios were plotted against pJNK/JNK ratios, arranged in ascending order into three equally sized groups (low, medium and high). High expression of VANGL2 protein is correlated to high levels of phosphorylated JNK. Box and whisker plots show the median value and interquartile ranges. The Kruskal–Wallis test was used for comparison of the median levels of expression. Statistically significant differences are indicated (*P≤0.05; **P≤0.01). (**e**) Treatment of the indicated PDX-derived cells (PDX. DC-2, −27, −13 and −26) with a Tat-conjugated JNK inhibitor (Tat-JIP at 10 μM) during 48 h led to greater reduction in cell proliferation of VANGL2^high^/pJNK^high^ than VANGL2^low^/pJNK^low^ PDX-derived cells. Comparisons use Tukey's multiple comparisons test. NS, not significant. Data are representative of three independent experiments; *P≤0.05; **P≤0.01; ***P≤0.001; ****P≤0.0001. (**f**) Treatment of the indicated PDX-derived cells (PDX. DC-2, −27, −13 and −26) with the p62^DN^ peptide (225 μM) but not with control scrambled peptide (225 μM) during 48 h resulted in decreased cell proliferation of VANGL2^high^/pJNK^high^, but not VANGL2^low^/pJNK^low^, PDX-derived cells. Data are representative of three independent experiments and statistical testing as stated in **e**.

p62/SQSTM1 has been associated with poor prognosis in various types of cancer, and functions as an oncogenic adaptor protein^18^,^19^. We next addressed whether the VANGL2–p62/SQSTM1 complex and its associated JNK signalling pathway have a significance in tumour progression. Our patient breast cancer data show that upregulation of *p62/SQSTM1* tended to be associated with poor MFS (5-year MFS 54% versus 62%, log-rank test, *P*=0.088; not shown) in agreement with published work^18^,^19^. A multivariate analysis of *VANGL2* and *p62/SQSTM1* expression based on the Akaike information criterion highlighted cooperation between these two genes in prognostic term (Fig. 7a). The functional cooperation between VANGL2 and p62/SQSTM1 was confirmed *in vitro* in anchorage-independent assays by the apparent increase in tumorigenicity of breast cancer cells ectopically co-expressing VANGL2 and p62/SQSTM1 (Fig. 7b). In addition, expression of the dominant-negative GFP-p62^DN^ in SUM149 cells led to a significant decrease in cell migration (Supplementary Fig. 7), suggesting that this effect is due to the destabilization of the VANGL2–p62/SQSTM1 complex. To evaluate whether the p62^DN^ sequence alone is able to elicit a similar effect, the peptide was fused to a fluorescent cell-penetrating fluorescein isothiocyanate (FITC)-Tat peptide (FITC-Tat-p62^DN^). This chimeric peptide was as efficient as the p62^DN^ peptide devoid of FITC-Tat to inhibit the VANGL2–p62/SQSTM1 interaction (Fig. 3e) in co-immunoprecipitation assays using similar concentration ranges (Supplementary Fig. 7). When membrane-permeant peptides were added to the culture media of breast cancer cells, a specific decrease in cell proliferation was detected in high VANGL2-expressing cells (SUM149 and HCC1806) with FITC-Tat-p62^DN^ peptide, but not with the control peptide (Supplementary Fig. 7). Interestingly, when the same experiment was carried out on the low VANGL2-expressing T47D cell line, neither FITC-Tat-p62^DN^ nor the control peptide had any significant effect on the proliferation rate. These results suggest that the effect of FITC-Tat-p62^DN^ depends on the expression of VANGL2 and of its interaction with p62/SQSTM1, and has no intrinsic antiproliferative properties. To challenge this point in experiments akin to pathological situations, we used recently characterized breast cancer PDXs that retain the histopathological and molecular features of the original primary tumours^34^. Thirty breast cancer PDXs were characterized for VANGL2 and p62/SQSTM1 expression as well as JNK activation levels with western blot analysis (Fig. 7c). While variations of the levels of p62/SQSTM1 and JNK appeared modest, those of VANGL2 as well as phospho-JNK were more striking. We quantified the expression levels of VANGL2, tubulin, JNK and phospho-JNK in our PDX series and plotted the VANGL2/tubulin against the phospho-JNK/total JNK ratios (Fig. 7d). A correlation was found between VANGL2 and phospho-JNK signal intensities (*R*^2^=0.494, where *R*^2^ is the coefficient of determination), and differences between low, medium and high expression of phospho-JNK with VANGL2 expression were observed. A similar analysis revealed no correlation between p62/SQSTM1 and phospho-JNK signal intensities (*R*^2^=0.043).

The presence of the VANGL2–p62/SQSTM1 complex in breast cancer PDX protein extracts was confirmed by VANGL2 immunoprecipitation followed by western blot analysis (Supplementary Fig. 7). We then addressed whether the VANGL2–p62/SQSTM1 complex and associated JNK activation contribute to cell proliferation. To this aim, the sensitivity to membrane-permeant peptides inhibiting either JNK activation (Tat-JIP) or destabilizing the VANGL2–p62/SQSTM1 interaction (FITC-Tat-p62^DN^) was evaluated in a proliferation assay on four breast cancer PDX-derived cell lines expressing high (PDX 13 and 26) or low (PDX 2 and 27) levels of VANGL2 that correlated with phospho-JNK contents (Supplementary Fig. 7). PDX-derived cells with a VANGL2^high^/pJNK^high^ phenotype were found to be more proliferative and more sensitive to JNK inhibition than VANGL2^low^/pJNK^low^ PDX-derived cells (not shown). Indeed, as illustrated in Fig. 7e, treatment with the Tat-JIP peptide led to a stronger reduction in cell proliferation in VANGL2^high^/pJNK^high^ cells (60–70% reduction) than in VANGL2^low^/pJNK^low^ cells (20–30% reduction). Moreover, only VANGL2^high^/pJNK^high^ PDX-derived cells showed a significant decrease in cell proliferation and JNK phosphorylation on incubation with the FITC-Tat-p62^DN^, whereas the control peptide had no effect (Fig. 7f and Supplementary Fig. 7). Taken together, these data show that overexpression of VANGL2 in breast cancer cells correlates with JNK activation and cell proliferation, which is sensitive to inhibition of the VANGL2–p62/SQSTM1 interaction.

## Discussion

The canonical β-catenin-dependent Wnt signalling is a molecularly well-defined pathway known to be involved in breast cancer progression for many years^3^. In contrast, the role of the non-canonical Wnt/PCP pathway in this disease and the underlying molecular mechanisms are much less well understood.

Here we find that the non-canonical Wnt/PCP *VANGL2* gene is linked to a basal signature and is overexpressed in breast cancers with poor prognosis (Fig. 1). In our data set, 23% of breast cancers showed *VANGL2* mRNA upregulation with a good correlation with gene amplification. In agreement with our findings, a recent study concluded that *VANGL2* is frequently overexpressed in endocrine-related cancers, among which 24% are invasive breast carcinoma^35^. In contrast, upregulation of the paralogue gene *VANGL1* is observed in less than 5% of invasive breast carcinoma and is associated with increased relapse rate and reduced survival in luminal breast cancers, suggesting a differential contribution of the *VANGL* family members to breast tumorigenesis^8^,^35^.

Until now, functional studies gathered in breast cancer cells on the VANGL family have focused on VANGL1 and have led to the conclusion that this Wnt/PCP protein plays a role in cell motility and invasiveness, with no obvious involvement in tumour growth^7^,^8^. The closely related VANGL2 protein is also endowed with promigratory functions in chronic lymphoid leukaemia, epithelial cells and fibroblasts^23^,^36^. Using cell culture and murine assays, we now demonstrate that VANGL2 is involved in breast cancer cell migration, anchorage-dependent and -independent cell proliferation as well as tumour growth (Fig. 2). Results obtained in cultured cell lines were confirmed in breast cancer PDX-derived cells that grow at higher cell proliferation rate in the context of VANGL2 overexpression (Fig. 7). These results suggest that VANGL2, a core Wnt/PCP component, participates in the growth of breast cancer cells.

To gain insight into the molecular mechanism underlying the role of VANGL2 in this disease, we purified the VANGL2 protein complex and identified p62/SQSTM1, a PB1 domain-containing protein with oncogenic functions as a VANGL2-binding partner (Fig. 3). We demonstrate the involvement of this scaffold protein in VANGL2–JNK signalling. Indeed, p62/SQSTM1 directly interacts with the carboxyl-terminal region of VANGL2 and recruits JNK, a downstream component of the Wnt/PCP pathway^3^, thus promoting its phosphorylation (Fig. 5). The results obtained in breast cancer cells and in *Xenopus* show the existence of an evolutionarily conserved WNT5A–VANGL2–p62/SQSTM1–JNK signalling pathway (Fig. 6).

In breast cancer PDXs, a good correlation was found between levels of VANGL2 and phosphorylated JNK (Fig. 7). Recent data have linked cell polarity, JNK and cell proliferation in breast tumours. Indeed, loss of PAR3, a major regulator of apicobasal polarity, leads to JNK-mediated proliferation of transformed mammary cells and tumour development in a RAC1-dependent manner^37^. Because VANGL2 binds to RAC1 (ref. 38), it is possible that this small GTPase contributes to the VANGL2–p62/SQSTM1–JNK pathway in breast cancer cells. The involvement of the scaffold JNK-interacting protein (JIP1) in JNK activation is also likely, as VANGL2^high^/pJNK^high^ PDX-derived cells were sensitive to TAT-JIP, a peptidic inhibitor derived from JIP1 (Fig. 7). JIP1 coordinates JNK signalling by recruiting MAPK Kinases (MKKs), such as MKK4/MKK7, and JNK^39^. As JNK constitutively binds to the VANGL2–p62/SQSTM1 complex and becomes phosphorylated solely on stimulation, we propose that JIP1/MKKs are involved in this activation step. Other studies have assigned a role of Wnt/PCP deregulation in the activation of the Hippo and FYN-STAT3 pathways, which, respectively, promote breast cancer stem cell renewal and EMT^9^,^40^. Thus, key steps of breast cancer development (initiation, growth and spread) are likely controlled by different Wnt/PCP-related signalling events.

In polarized epithelial tissues, VANGL2 is present in endocytic compartments^28^ and at the plasma membrane where it interacts in *trans* with Frizzled receptors^41^,^42^. We confirmed the plasma membrane localization of VANGL2 in polarized IMCD3 cells (Fig. 4). In SKBR7 breast cancer cells, we found a poor recruitment of VANGL2 at the plasma membrane, likely due to the loss of cell junctions. In agreement with this hypothesis, loss of epithelial cell–cell contacts following EGTA treatment redirected VANGL2 to endosomal compartments and increased its interaction with p62/SQSTM1 (Fig. 4). In addition, consistent with this idea, the interaction between VANGL2 and p62/SQSTM1 is required for convergence extension of nonepithelial tissues in *Xenopus* embryos (Fig. 6). Loss of cell polarity commonly observed in carcinomas and during EMT could thus favour the formation of VANGL2–p62/SQSTM1 complex and the subsequent JNK-mediated cell proliferation. Interestingly, it was recently reported that p62/SQSTM1 regulates levels of junctional proteins and that its overexpression induces EMT. Upregulation of p62/SQSTM1 may thus have multiple outcomes, each of which contributes to breast cancer aggressiveness^19^,^20^.

The colocalization of VANGL2–p62/SQSTM1 in late endosomes and its role in JNK activation is reminiscent of previous work reporting JNK signalling in endosomes. Indeed, phosphorylated JNK has been recently localized in LAMP1-positive late endosomes together with its signalling partners, the B-cell receptor and Ezrin^43^. Interestingly, p62/SQSTM1 was shown to recruit mTOR to the lysosomal membrane, which stands as an important hub for this pathway^44^. We thus suggest that VANGL2–p62/SQSTM1-JNK signalling occurs in part in late endosomal compartments.

The basal breast cancer subtype is associated with higher mortality than the other subtypes because of the poor success of current therapies. Our finding that VANGL2–p62/SQSTM1–JNK signalling is upregulated in this disease leads us to consider its components as potential therapeutic targets. This is actually the case for JNK that has become a target for drug development in metabolic diseases, inflammation and cancer. However, because it plays a pivotal role in numerous biological functions, inhibition of JNK is predicted to have significant side effects^45^. p62/SQSTM1 could represent an alternative therapeutic target, as its degradation can be triggered by induction of autophagy using rapamycin-like drugs such as everolimus. According to our results, this strategy should mimic the results obtained with p62/SQSTM1 siRNA and impair JNK signalling in breast cancer cells (Supplementary Fig. 5). Promising results have been recently obtained with everolimus in preclinical assays^46^ and clinical trials in basal breast cancers^47^. On the other hand, autophagy inhibition may also enhance therapeutic response to chemotherapy^48^. The potential of targeting autophagy in basal breast cancer deserves further investigation. Over the course of our study, we have identified a peptide able to destabilize the VANGL2–p62/SQSTM1 complex, and to impair JNK signalling and cell proliferation of breast cancer cells. This blocking strategy may represent a more specific, and possibly, less harmful alternative to inhibit VANGL2–p62/SQSTM1–JNK signalling in VANGL2-overexpressing breast cancers.

## Methods

### Cell culture and cell transfection and knockdown experiments

All epithelial breast cancer cell lines (T47D, HCC1806 and SUM149) were purchased and grown in accordance with American Type Culture Collection recommendations. SKBR7 (National Center for Biotechnology Information (NCBI) Gene Expression Omnibus (GEO): GSM75171 record,epithelial breast tumour cell line SKBR7_b39_s31 (*Homo sapiens*)) cells were grown in RPMI or F12 medium supplemented with 100 U ml^−1^ of penicillin, 100 mg ml^−1^ of streptomycin and 10% heat-inactivated fetal bovine serum. All cell lines were tested negative for mycoplasma contamination. Murine epithelial IMCD3 cell line was cultured in DMEM/F12 growth media. T47D cells were transfected with pEGFP using Lipofectamine 2000 reagent according to the manufacturer's instructions (Invitrogen). SUM149 cell nucleofection was performed according to the manufacturer's protocol (Amaxa). Nutrient deprivation was achieved by incubating cells with Earle's balanced salt solution (EBSS) or Hank's balanced salt solution (HBSS) (GIBCO). Bafilomycin A1 and puromycin (Sigma) were used at the specified concentrations and for the indicated time. Rapamycin (Calbiochem) was used at the specified concentration and for the indicated time.

### Animal models

All mouse husbandry and experimental procedures were performed in accordance with the protocols approved and in compliance with policies approved by the local Committee for Animal Experimentation (CAE of Provence number 14) of Marseille, France (2–091009). NSG mice were obtained from Charles Rivers (UK). Mice were housed under sterile conditions with sterilized food and water provided *ad libitum* and were maintained on a 12-h light and 12-h dark cycle. SUM149 (5 × 10^6^) and HCC1806 cells (1 × 10^6^) were subcutaneously inoculated into the right flank of 4–6-week-old female NSG mice. Tumour growth was monitored by measuring with a digital caliper and by calculating tumour volume (length × width^2^ × *π*/6). No blinding was used. For *in vivo* VANGL2-overexpression tumorigenesis experiments, COMMA-D cells were injected into cleared fat pads of 4-week-old female BALB/c as previously described^24^. Mice were examined weekly for palpable tumours. Once palpable tumours were found, they were measured with calipers weekly. Mice were killed when the calculated tumour volume reached 1 cm.

### *Xenopus* experiments

*Xenopus* embryo collection, microinjection, whole-mount *in situ* hybridization, animal cap assays and *xPAPC* reverse transcriptase–quantitative PCR conditions have all been described previously^49^. The riboprobe against *Xenopus p62/SQSTM1* was derived from the commercial plasmid IMAGE clone 8541876 (Biovalley, France). The MOs used in this study were purchased from GeneTools. Their respective sequences are as follows: p62/SQSTM1: 5′- AGGCTTTCACGGTGACCGTCATGTT -3′; p62/SQSTM1 5mis-MO: 5′- AGCCTTTCACCGTCACCCTCATCTT -3′; p62/SQSTM1 MO2: 5′- CAATGCACCTGACAGGCGGTGAT -3′.

### *VANGL2* mRNA expression analysis in breast cancer samples

Samples of human origin and associated data were obtained from the IPC/CRCM Tumour Bank that operates under authorization # AC-2007-33 granted by the French Ministry of Research (Ministère de la Recherche et de l'Enseignement Supérieur). Before scientific use of samples and data, patients were appropriately informed and asked to consent in writing, in compliance with French and European regulations. The project was approved by the IPC Institutional Review Board (Comité d'Orientation Stratégique).

To determine *VANGL2* mRNA expression in breast cancer and NB, we analysed gene expression data generated by our laboratory coupled with publicly available data sets. Our series included tumour tissues from 330 patients with invasive adenocarcinoma and four pools of NB tissue samples (11 healthy women). The 14 public data sets comprising one probe set representing *VANGL2* were collected from the NCBI/Genbank GEO database, the European Bioinformatics Institute ArrayExpress database or at the authors' websites (Supplementary Table 3). This resulted in a total of 2,687 nonredundant invasive breast cancers with *VANGL2* mRNA expression and histoclinical data available for analysis. For the Agilent-based data sets, we applied quantile normalization to available processed data. Regarding the Affymetrix-based data sets, we used Robust Multichip Average^50^ with the nonparametric quartile algorithm as normalization parameter. Before analysis, expression level for each tumour was centred by the average expression level of the four NB samples. To be comparable across data sets and to exclude bias from population heterogeneity, *VANGL2* expression levels were standardized within each data set using the luminal A population as reference. All steps were performed in R using Bioconductor and associated packages. *VANGL2* upregulation in a tumour was defined by a ratio tumour/NB ⩾2, downregulation by a ratio ≤0.5 and no deregulation by a ratio >0.5 and <2. The molecular subtypes of tumours were defined using the PAM50 Predictor^51^. To investigate the mRNA expression of *VANGL2* in mammary cell lines, we explored our previously published gene expression data of 35 cell lines, including SKBR7 and SUM149, profiled using oligonucleotide microarrays^22^.

### *VANGL2* DNA copy number analysis in breast cancer

We analysed aCGH data of 208 breast cancers profiled in our laboratory using 244-K CGH microarrays (Hu-244A, Agilent Technologies) as previously described^52^. A pool of 13 normal male DNA had been used as reference. Extraction of data (log_2_ ratio) was carried out with CGH Analytics, whereas normalized and filtered log_2_ ratio was obtained from the ‘Feature Extraction' software (Agilent Technologies). The *VANGL2* locus at 1q21-q23 was analysed and copy number changes were characterized as reported previously^53^. Four probes (A_16_P35329154, A_16_P00175662, A_16_P35329208 and A_16_P35329224) matched the *VANGL2* gene on our Agilent chips. A DNA copy number alteration in a tumour was defined as 1.5-fold change as compared with normal DNA.

### Clinical statistical analysis

To compare the distribution according to categorical variables, we used the Fisher's exact test. The prognostic value of *VANGL2* deregulation was analysed in the subgroup of 1,208 nonstage IV patients with available follow-up. MFS was calculated from the date of diagnosis until the date of first metastatic relapse. Follow-up was measured from the date of diagnosis to the date of last news for patients without relapse. A total of 492 patients experienced metastatic relapse after a median time of 25 months from diagnosis, and 716 remained relapse-free with a median follow-up of 84 months. Survival was calculated using the Kaplan–Meier method and curves were compared with the log-rank test. The 5-year MFS was 62% (95% confidence interval, 59–65%) for the whole population. Univariate and multivariate survival analyses were performed using Cox regression analysis (Wald test). Variables tested in univariate analyses included patients' age at the time of diagnosis (≤50 versus >50 years) and pathological features including type, tumour size (pT: pT1 versus pT2–3), axillary lymph node status (pN: negative versus positive), grade (1 versus 2–3), IHC ER, PR and ERBB2 status (negative versus positive) and *VANGL2* deregulation (upregulation versus no upregulation). Variables with a *P* value<0.05 in univariate analysis were tested in multivariate analysis. To compare the prognostic value of *VANGL2* and *p62/SQSTM1/SQSTM* expression alone and in combination, we used multivariate analysis based on the choice of the best predictive model by minimization of the Akaike criterion (AIC) in a stepwise algorithm using both forward and backward directions. All statistical tests were two-sided at the 5% level of significance. Statistical analysis was performed in R and associated packages. We followed the reporting REcommendations for tumour MARKer prognostic studies (REMARK criteria)^54^.

### Experimental statistical analysis

Each data panel is representative of at least three independent replicate experiments. The s.d. or the s.e.m. are displayed and stated in each experiment. Further analysis of variance and subsequent *ad hoc* comparisons used Mann–Whitney, Student's *t*-tests or analysis of variance followed by Tukey's test (95% confidence intervals) using the GraphPad Prism software.

### Knockdown experiments

ShVANGL2 (targeting the 5′ untranslated repeat region 5′- GAGCGCTGCGGATACAAAG -3′ sequence and 5′- GTACCTTCGGACCACCAAG -3′) cloned into the pSUPER.retro.puro vector (OligoEngine) was transfected and selected in 0.5 μg ml^−1^ puromycin for stable reduction of VANGL2 protein expression in SUM149 cells. A shRNA control targeting the Luciferase protein (shLuc) was used in these experiments^55^. The VANGL2 siRNA (05: 5′- GCACCAAGAAGGUCCCAUU -3′, 06: 5′- GGGAUGAGCGGGAUGACAA -3′, 07: 5′- GACCGACACCGCUCUAAGA -3′, 08: 5′- GAUCCCAAGUCACACAAGU -3′), the p62/SQSTM1 siRNA (05: 5′- GAACAGATGGAGTCGGATA -3′, 06: 5′- GCATTGAAGTTGATATCGAT -3′, 07: 5′- CCACAGGGCTGAAGGAAGC -3′, 08: 5′- GGACCCATCTGTCTTCAAA -3′) and non-targeting siRNA controls are from Dharmacon. siRNA transfections were carried out with RNAiMAX (Invitrogen), as recommended by the supplier. Lentiviral vectors (PSL9-Venus-P62/SQSTM1-FGT and PSL9-ctrl) were used to produce highly concentrated virion particles (Vectorologie, Montpellier, France).

### Peptide production and peptide inhibitors

FITC-Tat-p62^DN^ (VDPSTGELQSLQMPESEGPSSLDPSQEGPTGLKEAALYPHLPP) and FITC-Tat-Scrambled (EQQPEGSLVPGSPSEDTHLLDQPPGLLSPAEPEMTSPLKASYG) peptides were synthesized by GenScript, USA. A FITC-Tat (YGRKKRRQRRR) cell-penetrating sequence was fused at the N-terminal end of peptides. JNK inhibitor II is from Calbiochem (Catalogue No. 420119).

### Antibodies

Monoclonal rat anti-VANGL2 (2G4 monoclonal antibody) was generated as described^21^. Anti-p62/SQSTM1 antibodies used in the study are as follows: mouse antibody (BD Biosciences 610833) or M01, clone 2C11, for EM experiments (Abnova: H00008878-M01) and guinea-pig polyclonal anti-p62 (Progen Biotechnik, GP62-C). Other antibodies used are as follows: rabbit antibody to LC3 (MBL Technology: PM036), rabbit antibody to GFP Abcam (ab290) and mouse antibody to α-tubulin (Sigma: B512), rabbit antibody to phospho-Thr183/Tyr185 SAPK/JNK (Cell Signalling: 9251) and SAPK/JNK (Cell Signalling: 9252), rabbit anti-β-catenin antibody (Santa Cruz Biotechnology: H102), mouse antibody to glyceraldehyde-3-phosphate dehydrogenase (Abcam: ab9484), mouse monoclonal ubiquitin antibody (Life Sensors: FK2 AB120) and secondary antibodies coupled to horseradish peroxidase (Jackson Immunoresearch). Alexa Fluor-conjugated antibodies were purchased from Molecular Probes, Invitrogen. Antibodies were used according to the recommendations of the manufacturers or associated references.

### Western blots and immunoprecipitation and GST pulldown assays

For western blots, conditions are indicated as shown above the gels. Cells were serum-starved for 24 h and either treated with the indicated inhibitors or controls, followed by stimulation with fetal calf serum or human recombinant Wnt5a (RD systems, 645-WN-010) for the indicated times. Protein extracts were separated using SDS–PAGE and probed as stated in the figures. For immunoprecipitation, after pre-clearing with agarose beads and incubation with antibodies, protein G-agarose beads were added to the lysates and bound immune complexes were recovered, washed three times in lysis buffer and separated on SDS–PAGE for western blot analysis^56^. GST pulldown assays were carried out as previously described. Full blots are provided for main and Supplementary Figures are provided in the Supplementary Data File.

### Cell migration assays

Cell migration was evaluated using 8-μm pore polycarbonate membrane transwell chambers (Corning Costar). The bottom side of the membrane was coated with 25 μg ml^−1^ rat-tail collagen I. Cells were serum-starved for 16 h and then plated in the top chamber. Medium with or without 10% fetal calf serum was added to the bottom chamber and cells were incubated for 12 h. Non-migrated cells were scraped from the top of the membrane. Migrated cells were fixed in 4% formaldehyde and stained with 0.1% crystal violet for counting. Alternatively, cells on the inserts were trypsinized and assessed by using the CellTiter-Glo Luminescent Cell Viability Assay as described by the manufacturer (Promega Corporation).

### Cell proliferation assays

Unless otherwise specified, all cell proliferation experiments used CellTiter-Glo Luminescent Cell Viability Assay. After plating cells on 96-well plates (3610, Corning Costar) in 100 μl media per well, cell proliferation activity was assessed at different times using the CellTiter-Glo Luminescent Cell Viability Assay as described by the manufacturer (Promega Corporation). Values are means and s.d. of three independent experiments.

### Measure of anchorage-independent cell proliferation

Anchorage-independent growth was measured in 96-well plates. The 6% agarose solution stock was diluted in SUM149 culture medium and 100 μl was used to make the 0.6% bottom layer in opaque with clear bottom 96-well assay plates. After the bottom layer was gelified, a serial twofold dilution of cells was mixed with complete culture media containing agarose at a final concentration of 0.3%. Fifty microlitres per well (corresponding to 250 and 500 cells) were added to the bottom layer. Cells were incubated for 21 days at 37 °C in 5% CO_2_ atmosphere. Unless otherwise stated, cell number was quantified using CellTiter-Glo reagent (Promega) as follows: 75 μl of CellTiter-Glo reagent (Promega) and luminescence was recorded after 10 min of incubation time using a microplate luminometer.

### Mass spectrometry analyses

Mass spectrometry analyses were carried out with LC-MS/MS using a LTQ-Velos-Orbitrap (Thermo Electron, Bremen, Germany) connected to a nanoLC Ultimate 3000 Rapid Separation Liquid chromatography system (Dionex, Sunnyvale, CA, USA). Five microlitres of peptide sample corresponding to one-sixth of the whole sample were injected for each analysis. After preconcentration and washing of the sample on a Dionex Acclaim PepMap100 C18 column (2 cm, 100 μm internal diameter (i.d.), 100A, 5 μm particle size), peptides were separated on a Dionex Acclaim PepMap RSLC C18 column (15 cm, 75 μm i.d., 100A, 2 μm particle size; Dionex, Amsterdam) at a flow rate of 300 nl min^−1^ using a two-step linear gradient (4–20% acetonitrile/H_2_O; 0.1% formic acid) for 90 min and 20–45% acetonitrile/H_2_O; 0.1% formic acid for 30 min. Separation of the peptides was monitored by a ultraviolet detector (absorption at 214 nm). The nanoLC was coupled to a nanospray source of a linear ion trap Orbitrap mass spectrometer (LTQ OrbitrapVelos, Thermo Electron). The linear trap quadrupole (LTQ) spray voltage was 1.4 kV and the capillary temperature was set at 275 °C. All samples were measured in a data-dependent acquisition mode. Each run was preceded by a blank MS run to monitor system background. The peptide masses are measured in a survey full scan (scan range 300–1,700 *m*/*z*, with 30 K full-width at half-maximum resolution at *m*/*z*=400, target AGC value of 1 × 10^6^ and maximum injection time of 200 ms). In parallel to the high-resolution full scan in the Orbitrap, the data-dependent CID scans of the 10 most intense precursor ions were fragmented and measured in the linear ion trap (normalized collision energy of 35%, activation time of 10 ms, target AGC value of 1 × 10^4^, maximum injection time of 100 ms and isolation window of 2 Da). The fragment ion masses are measured in the linear ion trap to have a maximum sensitivity and the maximum amount of MS/MS data. Dynamic exclusion was implemented with a repeat count of 1 and exclusion duration of 30 s.

Raw files generated from mass spectrometry analysis were processed with Proteome Discoverer 1.4 (Thermo Fisher Scientific). This software was used to search data via in-house Mascot server (version 2.2.03; Matrix Science Inc., London, UK) against the Human database subset (20,247 references) of the Swissprot 02 database. For the database search, the following settings were used: a maximum of one miscleavage, oxidation as a variable modification of methionine, carbamidomethylation as a fixed modification of cysteine and trypsin was set as the enzyme. A peptide mass tolerance of 6 p.p.m. and a fragment mass tolerance of 0.8 Da was used for search analysis. Only peptides with high stringency identity Mascot score (false discovery rate (FDR)<1%) were used for protein identification. For each immunoprecipitation experiment, three conditions were used to select specific protein complexes from experimental contaminants: positive immunoprecipitation with 2G4 anti-VANGL2 antibody or control beads alone or coupled to control isotypic antibody.

### Electron microscopy

Cells were fixed with 4% formaldehyde and 0.1% glutaraldehyde in 0.1 M phosphate buffer (pH 7.2), scraped and pelleted in 12% gelatin and infused with 2.3 M sucrose before mounting and freezing in lN_2_. Sections were cut at −110 °C, picked up with a 50:50 mixture of 2.3 M sucrose and 2% methyl cellulose and transferred on formvar/carbon-coated grids. Single labelling was performed with rat anti-VANGL2 and mouse-anti-p62 followed by secondary bridging antibodies (Sigma) and 10 nm protein A gold (CMC, Utrecht, the Netherlands). Double labelling against VANGL2 (1/50 antibody dilution) and p62 (1/300 antibody dilution) was performed sequentially with 0.1% glutaraldehyde as blocking step. Sections were observed at 80 kV in a JEOL-JEM 1230, micrographs recorded with a Morada charge-coupled device camera (SIS, Germany) and processed with the Photoshop software (Adobe).

## Additional information

**How to cite this article:** Puvirajesinghe, T. M. *et al*. Identification of p62/SQSTM1 as a component of non-canonical Wnt VANGL2–JNK signalling in breast cancer. *Nat. Commun.* 7:10318 doi: 10.1038/ncomms10318 (2016).

## Supplementary Material

Supplementary Information Supplementary Figures 1-7, Supplementary Tables 1-3 and Supplementary References

Supplementary Data 1 List of peptides obtained by LC-MS/MS in endogenous VANGL2 protein complexes (VANGL1, VANGL2, and p62/SQSTM1) is provided as an Excel file. VANGL2 protein complexes were immunoprecipitated using VANGL2 G4 antibody. Control immunoprecipitation carried out using isotypic Ig2a antibody or in absence of antibodies were added to identify specific VANGL2 partners from unspecific contaminating proteins. Protein identification was performed using LC-MS/MS analysis. Full Proteome Discover (ThermoFisherScientific) results obtained following data search using mascot engine against the human protein sequences of the Swissprot database are presented. Only peptides identified with higher mascot score (high stringency) were used for protein identification. False discovery rate for peptide identification was <1 %. Proteins were initially separated using a precast gradient gel. Only proteins located in the gel slice corresponding to 60 kDa were included in the mass spectrometry analysis.

Supplementary Data 2 List of peptides obtained in the VANGL2 protein complex by LC-MS/MS is provided as an Excel file. For mass spectrometry analysis, the immunoprecipitated protein samples were loaded on a 4-12 % Bis-Tris acrylamide gel but gel migration was stopped as soon as proteins stacked into a single band. Protein containing bands were stained with Imperial Blue (Pierce), cut from the gel and digested with high sequencing grade trypsin (Promega). Analysis was carried out as detailed in the legend to Supplementary Data 1.

## Figures and Tables

**Table 1:** LC-MS/MS using LTQ-Velos-Orbitrap mass spectrometry analysis of proteins co-immunoprecipitated with VANGL2 in SUM149 cell extracts.

**Protein**	**Swissprot name, accession number**	**Sequence coverage %**	**Mascot score**	**No. of identified peptides**	**Number of unique peptides**
VANGL2	VANGL2_HUMAN, Q9ULK5	31	981	42	13
VANGL1	VANGL1_HUMAN, Q8TAA9	26	1078	36	10
p62	SQSTM1_HUMAN, Q13501	66	2419	65	14

Proteins identified include known protein–protein interactions, VANGL1 and novel (p62/SQSTM1) binding partners. Protein sequence coverage, Mascot score, number of peptide-spectrum matches (identified peptides) and number of unique identified peptides are indicated for each protein. The experiment was repeated three times. Full experimental protein lists are shown in Supplementary Data 1.
