# Supplementary material for: Identification of p62/SQSTM1 as a component of non-canonical Wnt VANGL2–JNK signalling in breast cancer
Source: Nat Commun. 2016 Jan 12;7:10318. doi: 10.1038/ncomms10318 (PMC4729931; doi:10.1038/ncomms10318)
Supplement: Supplementary Information — Supplementary Figures 1-7, Supplementary Tables 1-3 and Supplementary References [file ncomms10318-s1.pdf]

Supplementary Fig. 1 related to Fig. 1: VANGL2 is overexpressed in basal and mesenchymal cell lines.

Hierarchical clustering of 35 mammary cell lines and 13,976 genes/ESTs based on mRNA expression levels. Each row represents a gene and each column represents a sample. The expression level of each gene in a single sample is relative to its median abundance across all samples and is depicted according to a colour scale shown at the bottom. Red and green indicate expression levels respectively above and below the median. The magnitude of deviation from the median is represented by the colour saturation. The dendrogram of samples (above matrix) represents overall similarities in gene expression profiles. Coloured bars to the left indicate the locations of five gene clusters of interest. The “basal” gene cluster (red bar) is zoomed on the right below the name of cell lines. The molecular subtype of cell lines is coloured as follows: blue for luminal, red for basal, orange for mesenchymal. Four cell lines are not attributed any subtype (white) according to Perou’s molecular subtype model. Expanded view of the “basal” gene cluster includes the *VANGL2* gene. Genes are referenced by their HUGO abbreviation as used in "Entrez Gene".

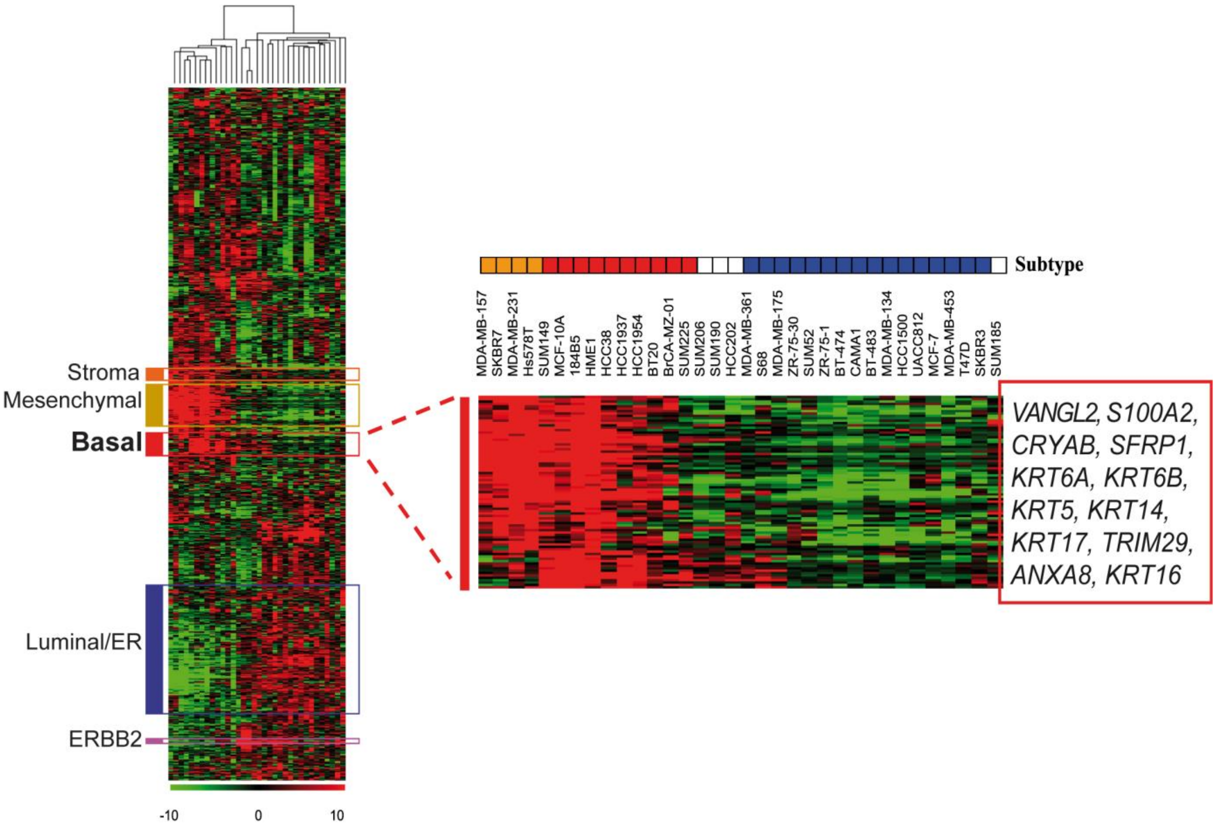

Supplementary Fig. 2 related to Fig. 2: Down-regulation of VANGL2 in SUM149 cells impairs tumour growth and cell migration. (a) Stable knockdown of VANGL2 in SUM149 cells with shVANGL2 (seq. 2) resulted in a decrease in tumour volume. SUM149 cells (5 × 10^6) were inoculated subcutaneously into the right flank of 4-6 weeks-old female NOD/SCID/γc null mice. The results represent an average of 2 experiments and corresponding to 11 and 13 mice, respectively, for shLuc and shVANGL2 transfected cell line. 2-way ANOVA value showing a statistical effect of the 2 conditions over time. (**b**) Knockdown of VANGL2 by shVANGL2 seq. 1 results in a decrease in anchorage-independent growth of SUM149 cells. Soft agar assay was carried out using 250 and 500 SUM149 cells. Cell number was quantified after 21 days of incubation. Results are the mean ± S.D. of three experiments; Student's *t*-test, **P*≤0.05. (**c** and **d**) SUM149 cells stably transfected with shLuc and shVANGL2 (seq. 1) were transiently transfected with GFP or GFP-VANGL2 and submitted to Boyden chamber assays using serum (FCS) as a chemo attractant. Decreased cell migration observed in the absence of VANGL2 was rescued with GFP-VANGL2 but not GFP. Results are the mean ± S.D. of three experiments. Asterisks represent significant differences relative to the control (****P*≤0.001 by Mann–Whitney *U* test). Data are represented as fold changes.

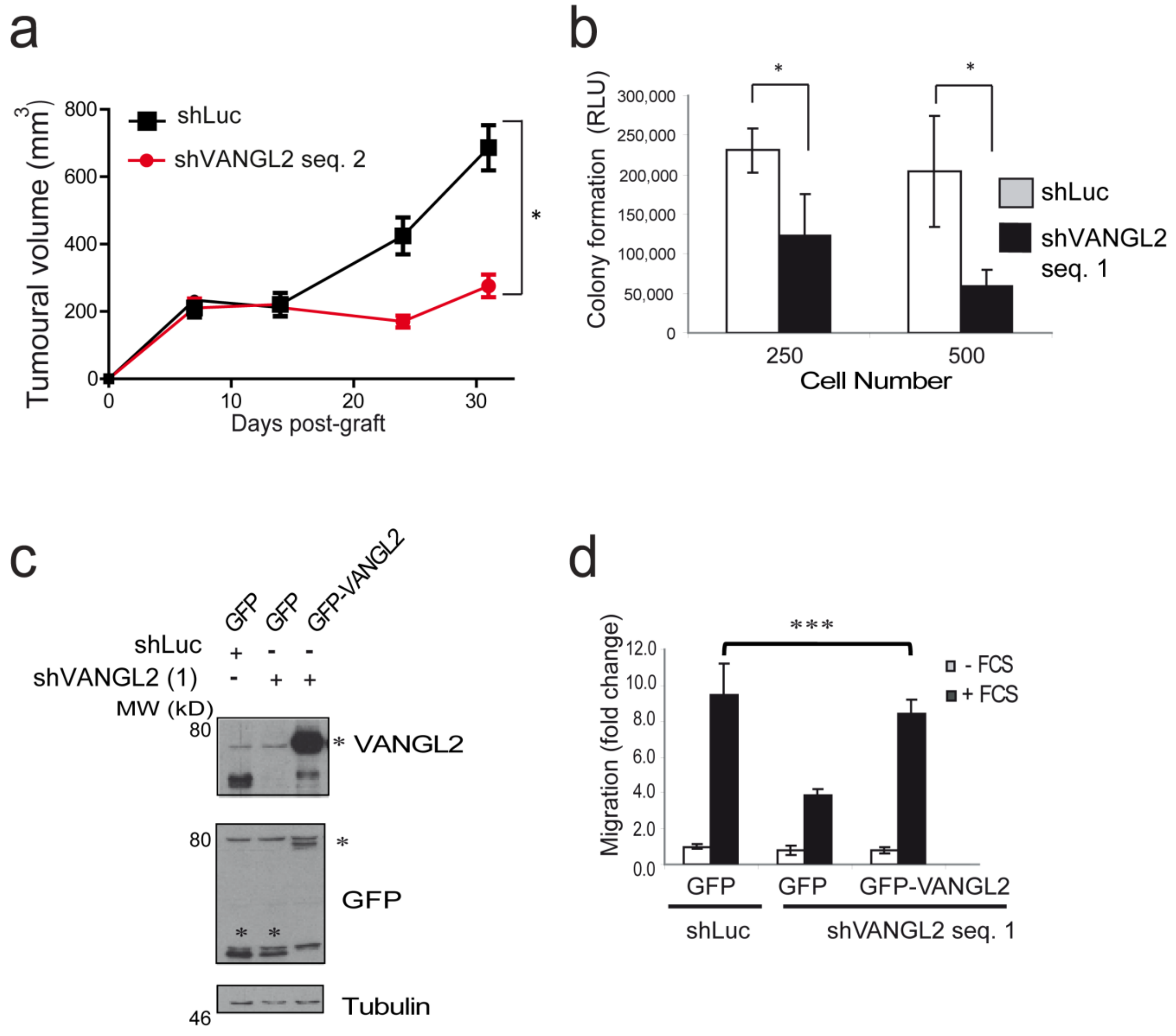

Supplementary Fig. 3 related to Fig. 3: Characterization of the VANGL2-p62/SQSTM1 interaction. (a) Immuno-purification of the VANGL2 complex in SKBR7, a basal breast cancer cell line, using anti-VANGL2 mAb for immunoprecipitation. **(b)** Same as (A) using a monoclonal anti-p62/SQSTM1 antibody for immunoprecipitation. Controls are isotype-matched antibodies. Experiments are representative of 3 experiments. **(c)** Schematic of VANGL2 containing two PDZ binding sites: S464 and TSV. **(d)** *In vitro* translation assays and pull-down experiments showed that VANGL2 binds to GST-p62/SQSTM1 via its C-terminal domain (CT). Deletion of the C-terminal TSV PDZ binding motif of VANGL2 corresponding to Scrib binding site or mutation of the Dishevelled PDZ binding site (S464N) did not affect the interaction with p62/SQSTM1. AR: autoradiography and CBB: Coomassie Brilliant Blue. Data are representative of 3 experiments.

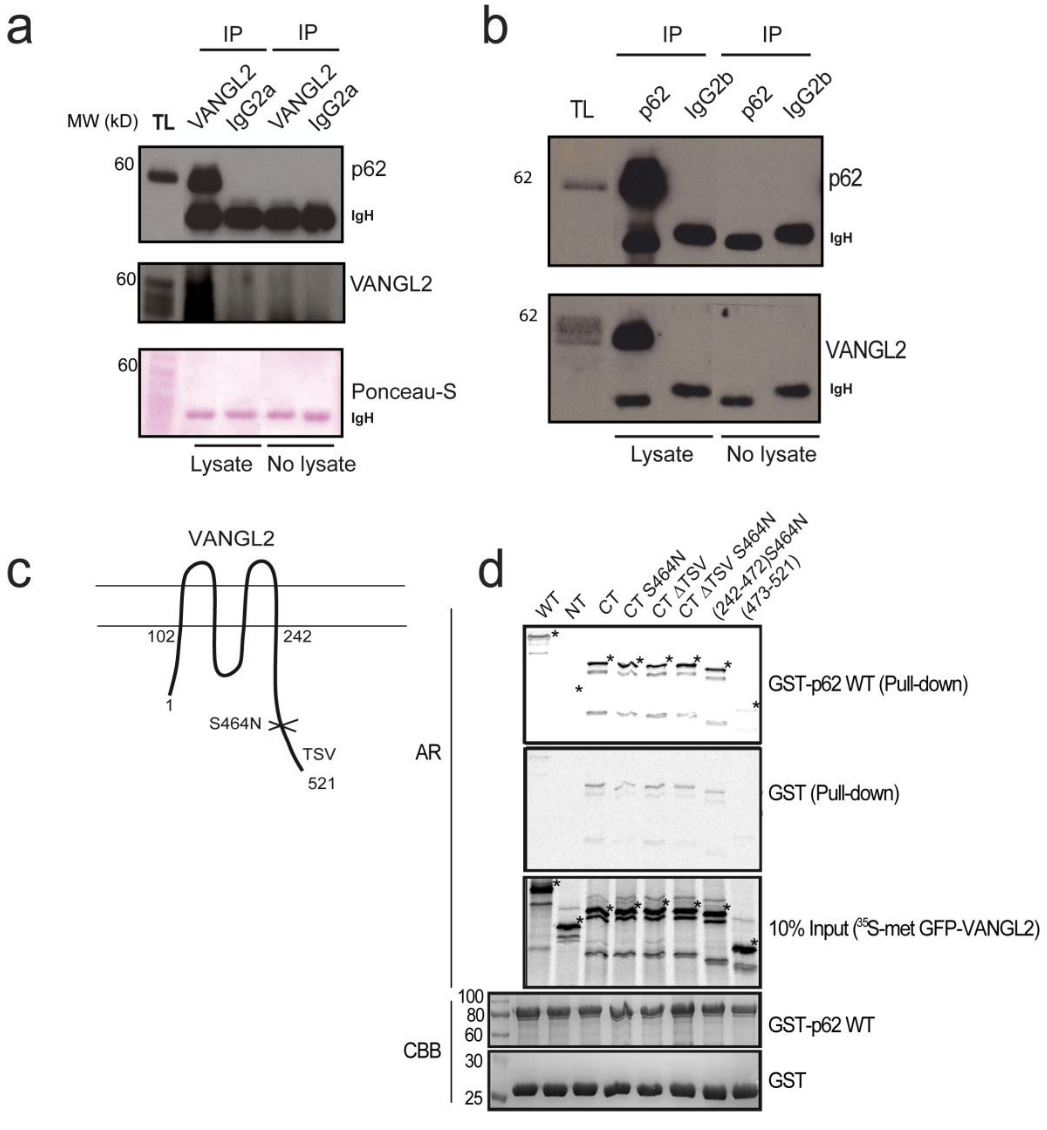

Supplementary Fig. 3 related to Fig. 3 (continued): Characterization of the VANGL2-p62/SQSTM1 interaction. (e) Schematic diagram of the various protein domains of the cytoplasmic multi-domain protein p62/SQSTM1 including a self-interacting N-terminal PB1 domain, Zn (zinc finger), TB (TRAF6-binding), LIR (LC3-interacting region), KIR (KEAP1-interacting region) and UBA (UBiquitin-Associated) domains **(f-i)** Characterization of the VANGL2 binding region of p62/SQSTM1 using deletion mutants of p62/SQSTM1**.** COS-7 cells transfected with GFP were used for immunoprecipitation experiments, and both lysates and immunoprecipitated products were analyzed by immunoblots with the indicated antibodies. Deletion mutants of GFP-p62/SQSTM1 identified regions important in the interaction with endogenous VANGL2. A p62/SQSTM1 PB1 mutant (GFP-p62 K7A/D69A) unable to self-assemble (monomeric p62)[2] still coimmunoprecipitated with VANGL2. Data are representative of 3 independent transfection experiments. Point mutations are indicated by blue arrows, deletions in amino acid sequence are indicated by red triangles. Interaction between endogenous VANGL2 and p62/SQSTM1 constructs indicated with (+), whilst no interaction was indicated with (-), as shown on the left of panel e.

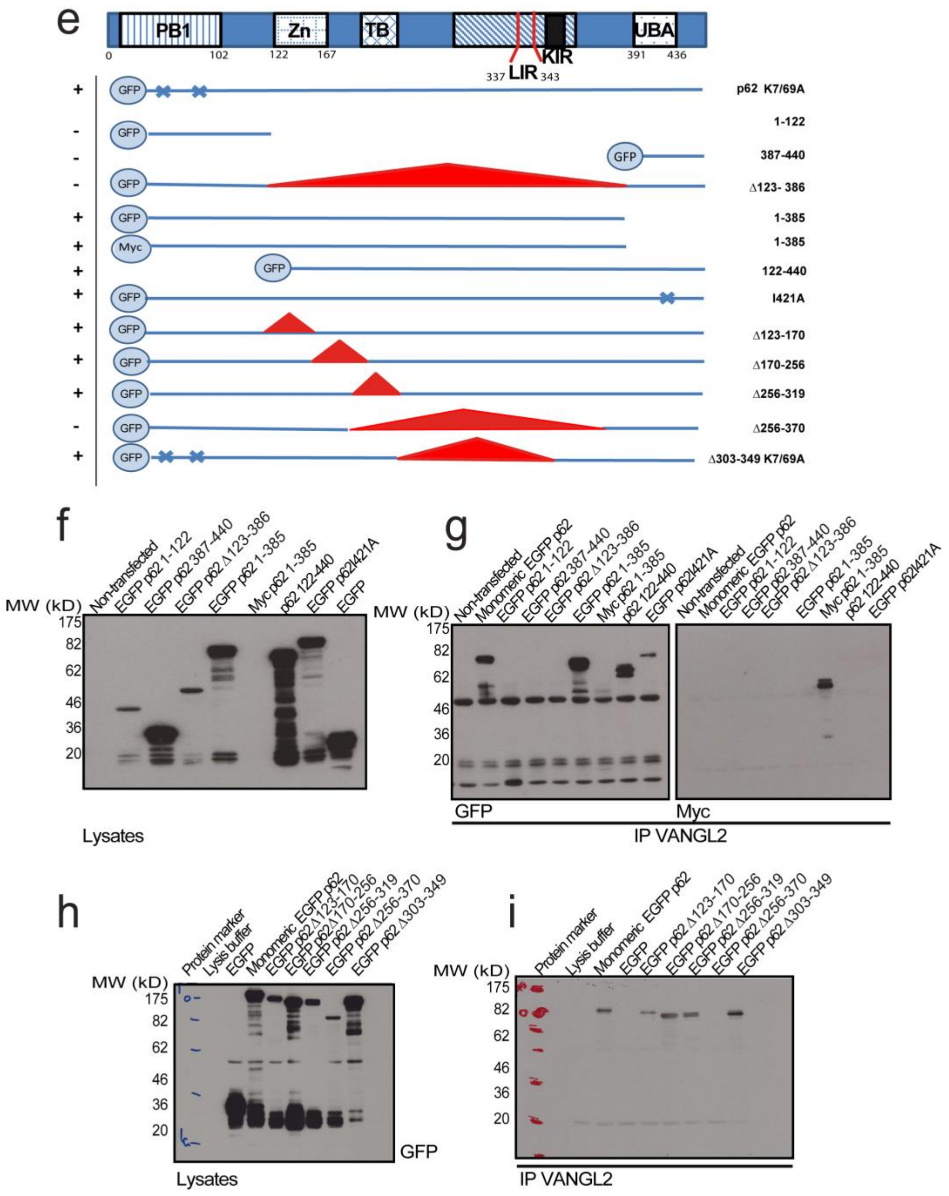

Supplementary Fig. 3 related to Fig. 3 (continued): Characterization of the VANGL2-p62/SQSTM1 interaction. (k). SUM149 cell lysates were incubated with the inhibitory peptide ($p62^{DN}$) or the control scrambled peptide (Ctrl peptide) at the indicated concentrations. VANGL2 was then immunoprecipitated using VANGL2G4 mAb. Presence of VANGL2, p62 and tubulin was detected by western blot. **(l)** Pre-incubation of SUM149 cell lysates with soluble competing peptide ($p62^{DN}$) or the control scrambled peptide (Ctrl peptide) was followed by immunoprecipitation of endogenous VANGL2 (using VANGL2G4 mAb), and mass spectrometry analysis of the complex. Numbers of identified peptides are indicated for the immunoprecipitated proteins in each experimental condition. At 50 µM, the $p62^{DN}$, but not the control peptide, efficiently competed the interaction between VANGL2 and p62/SQSTM1 but did not impair heterodimerization between VANGL2 and VANGL1. LC-MS/MS analysis representing abundance of each protein (number of peptides) in each experimental condition is provided in the table. PSM (Peptide Spectrum Matches) and distinct peptide sequences are indicated in Supplementary Data 2. **(m)** Western blot of lysates showed that different concentrations of p62/SQSTM1 and scrambled peptides added to lysates had no effect on total protein levels of VANGL2, p62/SQSTM1 and tubulin.

k

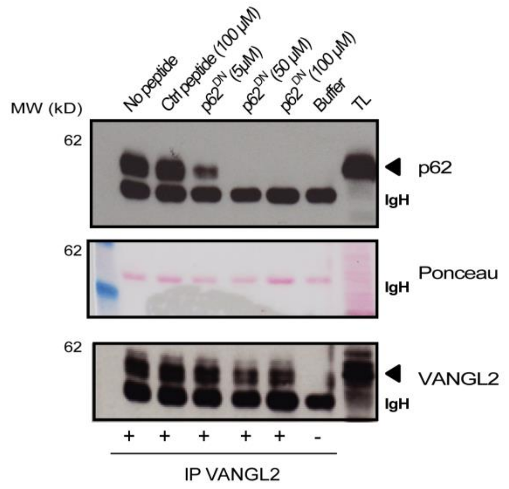

l

	No peptide	Ctrl peptide(50 µM)	$p62^{DN}$ peptide(5 µM)	$p62^{DN}$ peptide(50 µM)	Beads
VANGL2	27	28	20	12	0
VANGL1	18	22	19	11	0
p62	36	39	19	0	0

m

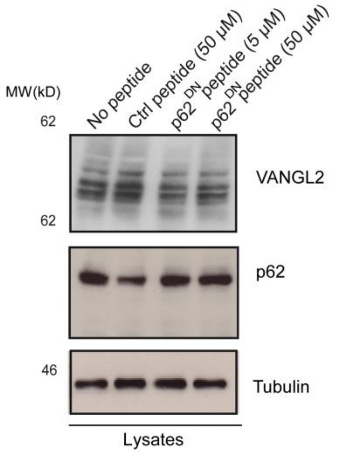

Supplementary Fig. 4 related to Fig. 4: VANGL2 colocalizes with p62/SQSTM1 in intracellular compartments and is not degraded by autophagy. Cells were cultured in nutrient-deprived medium and treated with 100 nM bafilomycin A1 (6 h) before fixation. Single and double labelling against VANGL2 (arrowheads in all Fig.s) and p62 (arrows in all Fig.s) as described in methods. Single labelling showed accumulations of both markers (VANGL2 in **a**, p62 in **b**) in vesicular structures resembling endosomes/amphisomes, as they contain both autophagic material and intraluminal vesicles typically found in multivesicular endosomes. Double labelling (**c**) confirmed colocalization in these structures. Scale bars 200 nm. **(d)** IMCD3 or SKBR7 cells were fixed, permeabilized, stained with antibodies directed VANGL2 (green), β-catenin (red) and DAPI, and immunofluorescence and confocal microscopy were performed. Scale bar (10 µm).

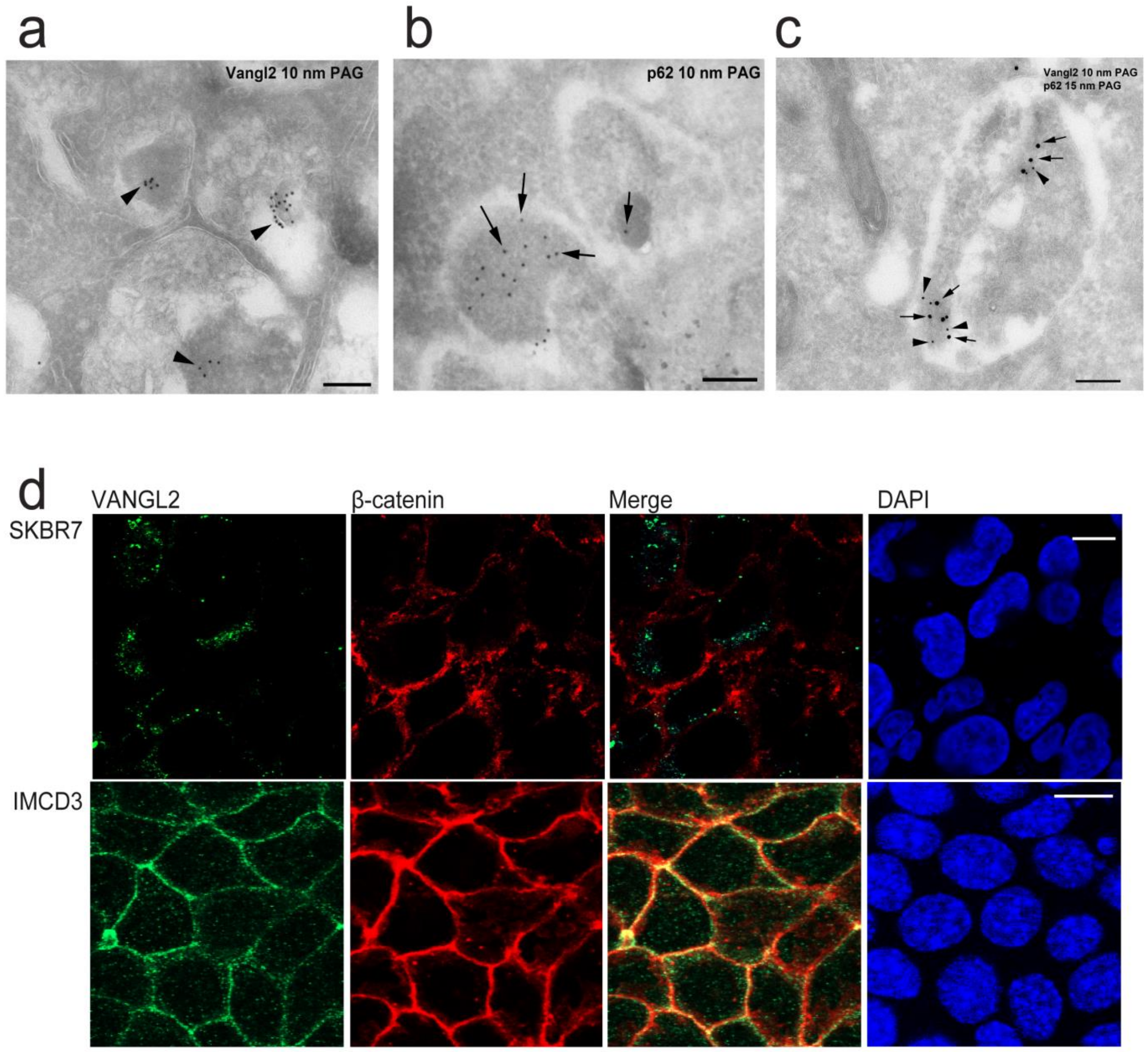

Supplementary Fig. 4 related to Fig. 4 (continued). VANGL2 colocalises with p62/SQSTM1 in intracellular compartments and is not degraded by autophagy. (e) SUM149 cells cultured in nutrient-deprived medium were treated with 100 nM bafilomycin A1 (6 h) or different concentrations of rapamycin (6 h). VANGL2, LC3 I and II levels were evaluated by western blot and quantified from fresh samples. Protein levels of VANGL2 remained unchanged (upper right graph). Data are representative of at least three independent experiments. **(f)** Mouse Embryonic Fibroblasts (MEFs) knock-out for p62/SQSTM1 (p62 KO MEFs) or wild-type (WT MEFs) were treated with amino acid deprivation for 6 hours or in full serum media in the presence and absence of bafilomycin A1. No change of VANGL2 protein levels was evidenced in absence of p62/SQSTM1. **(g)** SUM149 cells were treated with MG132, a proteasome inhibitor, for the indicated periods of time. MG132 treatment increased VANGL2 levels.

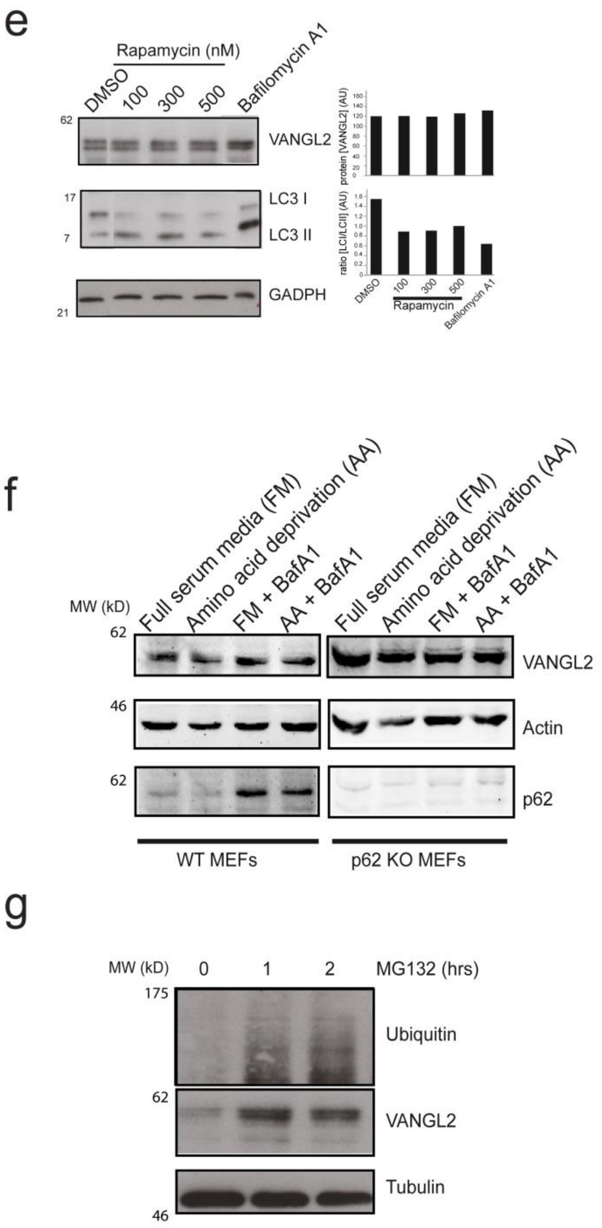

Supplementary Fig. 5 related to Fig. 5: Implication of p62/SQSTM1 in JNK signalling. **(a)** SUM149 cells were treated with the indicated p62 siRNAs and stimulated by Wnt5a (100 ng ml^{-1}) at different times. Proteins were revealed with the indicated antibodies by western blot (left) and the pJNK/JNK ratios were quantified (right). **(b)** GFP-p62^{DN} expressed in SUM149 cells, but not GFP alone, co-immunoprecipitated with VANGL2 and competed for the interaction between endogenous p62/SQSTM1 and VANGL2. **(c)** p62/SQSTM1 siRNA treatment of SUM149 cells abrogates the presence of JNK in the VANGL2 complex. Data is representative of results obtained from two different siRNAs. Red asterisks pinpoint IgH.

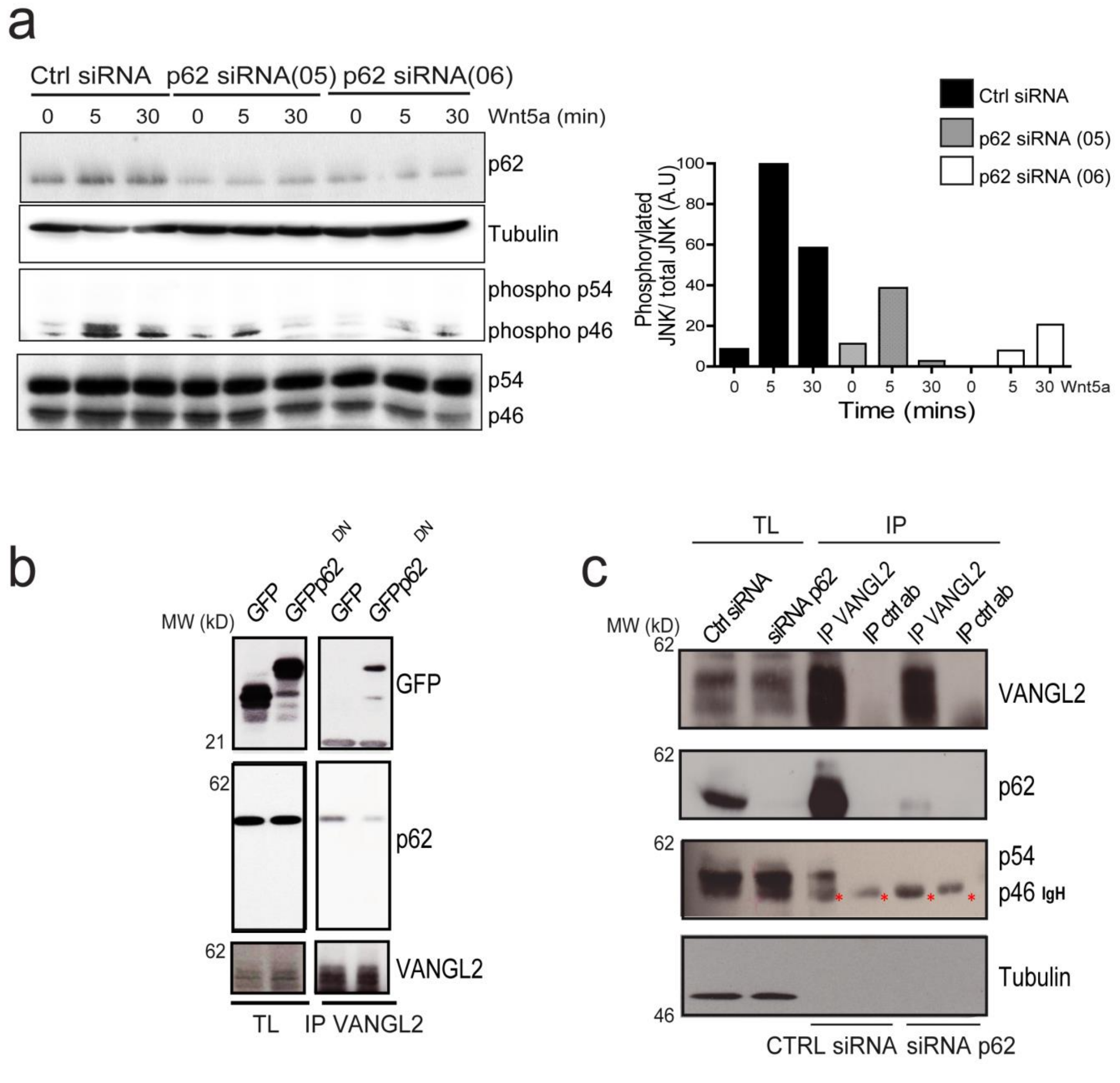

Supplementary Fig. 5 related to Fig. 5: Implication of p62/SQSTM1 in JNK signalling. (d) Proteins extracted from SUM149 cells treated or not with Wnt5a were immunoprecipitated with anti-VANGL2 antibody and blotted with the indicated antibodies. p62/SQSTM1, JNK and phosphorylated JNK were present in the VANGL2 complex.

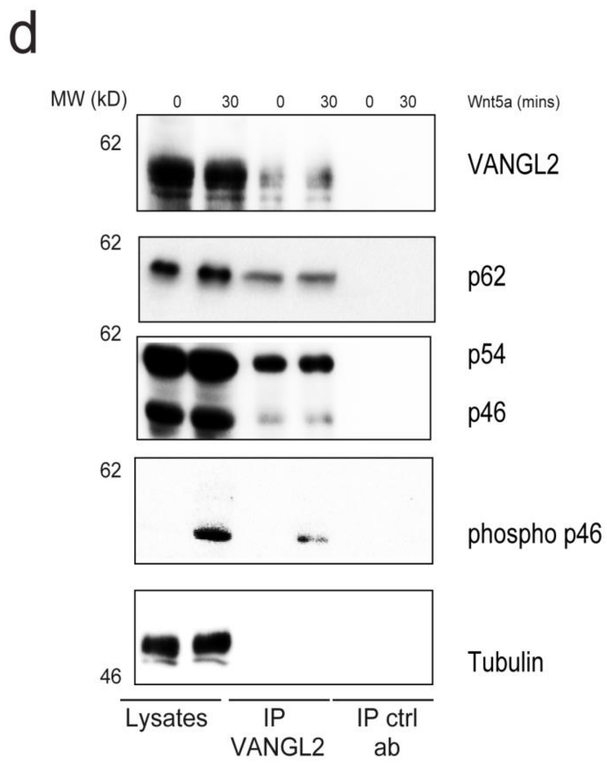

Supplementary Figure 6: p62/SQSTM1 homologues are conserved in the *Xenopus* genome and have overlapping expression patterns to VANGL2.

(**a**) Phylogenetic tree of p62/SQSTM1 sequences from different vertebrate species. (**b**) Synteny of the *p62/SQSTM1* genomic region. (**c**) Expression pattern of *p62/SQSTM1* in *Xenopus laevis* embryos (*In situ* hybridization).

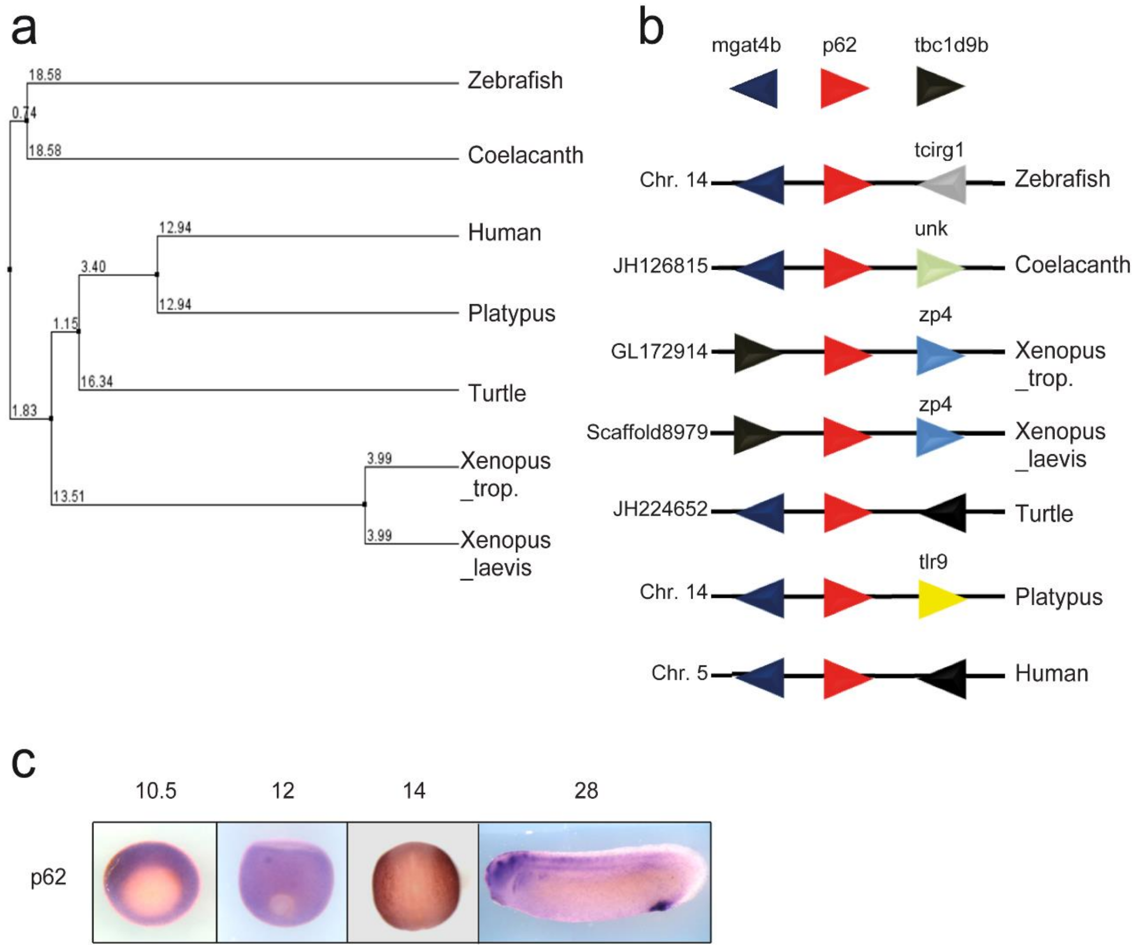

Supplementary Fig. 6 related to Fig. 6: p62/SQSTM1 is required for *Xenopus* morphogenesis and development. (**d**) Two-cell embryos were injected in each blastomere with 300, 200, 100, 50 and 20 ng of p62 morpholino carrying 5 mismatches (p62 5mis-MO). Amounts of p62 5mis-MO below 100 ng did not affect survival of injected embryos. (**e**,**f**) Two-cell embryos were injected as in (d). Morphology was analysed at tailbud stage. The p62 5mis-MO did not significantly alter embryonic morphogenesis and development at any of the doses tested. (**g**) Two-cell embryos were injected in each blastomere with 100 or 50 ng of p62 MO2, which has a sequence different from p62 MO. (**h**) Morphology was scored at tailbud stage. Note that both doses of p62 MO2 caused neural tube defects. (**i**) Embryos injected with p62 5mis-MO (n=116) and p62 MO2 (n=54) were processed for WISH analysis and stained with Sox2 probe to highlight neural tube closure defects. 94 % p62 5mis-MO embryos displayed a normal Sox2 staining. 79 % p62 MO2 embryos displayed enlarged Sox2 staining, typical of class II neural tube closure defects.

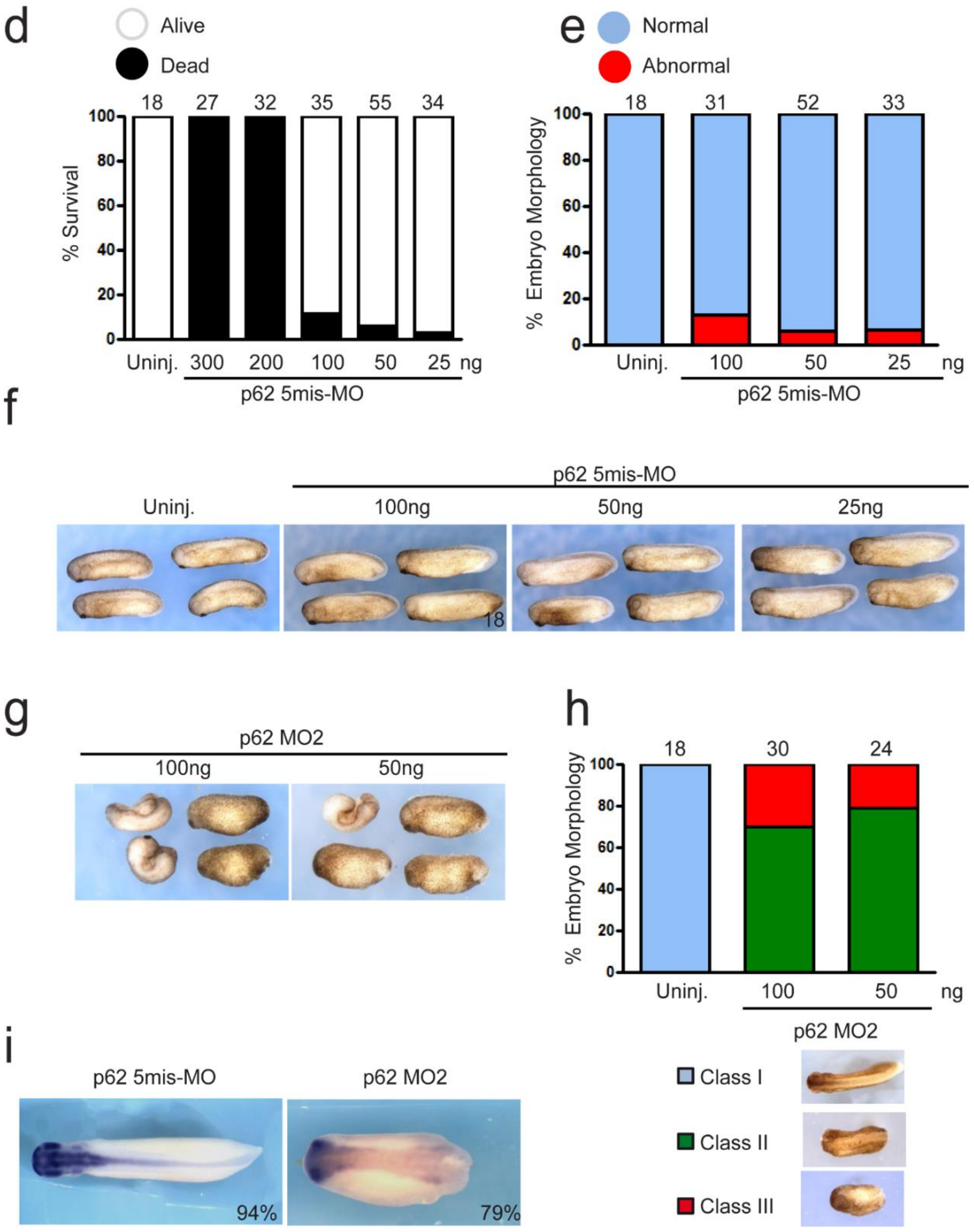

Supplementary Fig. 6 related to Fig. 6: *Xenopus* p62/SQSTM1 loss of function is rescued by human p62/SQSTM1. (**j**) Two-cell embryos were injected in each blastomere with 50 ng of p62 MO followed by injection at four-cell stage of synthetic human p62 mRNA (2.5 ng in each blastomere). Morphology was scored at tailbud stage, and embryos were then stained with Sox2 probe to highlight neural tube defects. The number of embryos analysed in each condition is displayed on the graph. (**k**) Quantification of blastopore rescue. Blastopore closure was estimated using the ratio of blastopore diameter at indicated stages to the mean of control (ctrl) blastopore diameter at st.10.5. Bars represent maximum and minimum values, and lines represent the mean. Approximately 30 embryos per condition were scored. (**l**) Embryos were processed for WISH analysis at mid-neurula and late gastrula stages. Average neural tube Length/Width ratio for embryos stained with Sox2 probe (n=11) or average notochord length for embryos stained with Xbra probe (n=11) were calculated. Five independent rescue experiments were scored. One-way-ANOVA with Tukey test (95 % confidence intervals) were applied (***P≤0.0005).

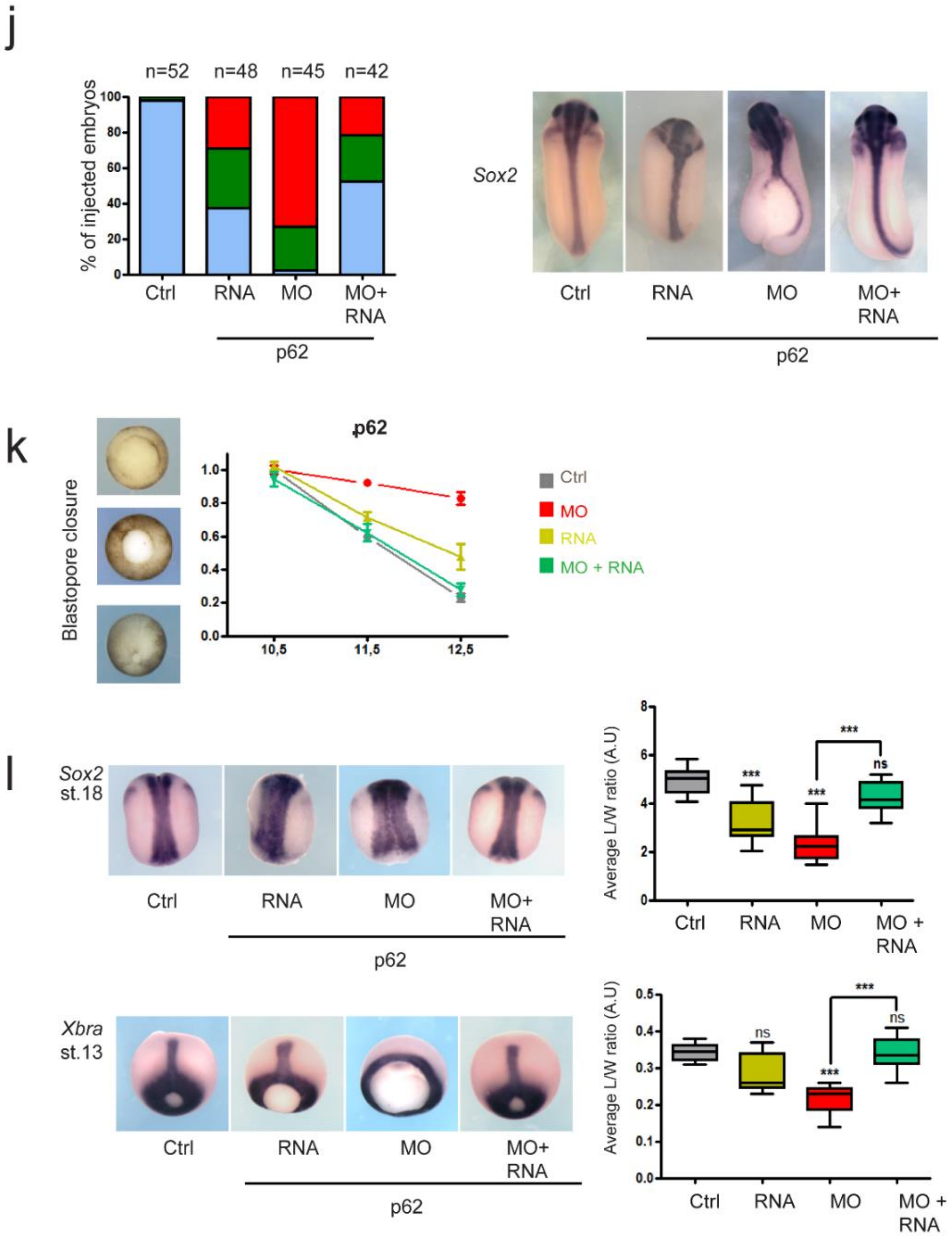

Supplementary Fig. 7 related to Fig. 7. Kaplan-Meier metastasis-free survival curves in breast cancer patients according to p62/SQSTM1 mRNA expression. (a) The 5-year MFS are 54 % (upregulation; N=106) and 62 % (absence of upregulation; N=1102).

a

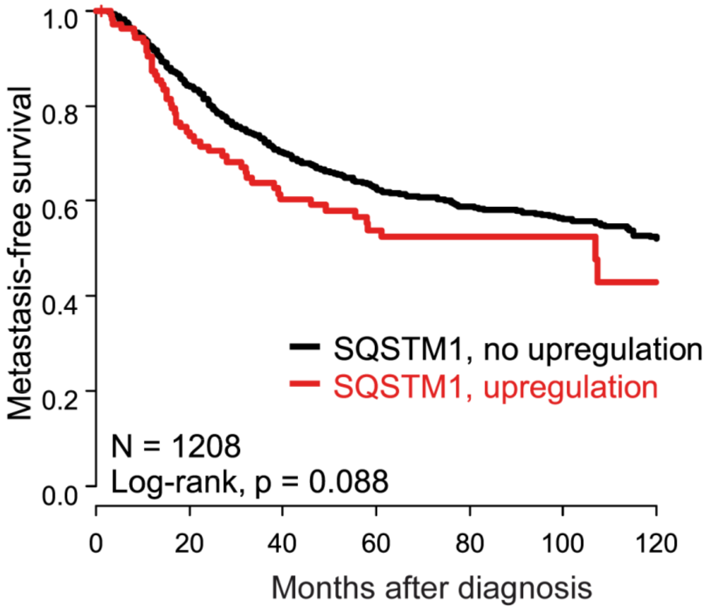

Supplementary Fig. 7 related to Fig. 7: The p62^{DN} peptide reduces cell migration, cell proliferation and JNK phosphorylation in breast cancer cells. (b) Expression of Venus-tag and Venus-p62^{DN} in SUM149 cells. Infection of SUM149 cells with highly concentrated viral stocks of Venus-p62^{DN} and control (Ctrl) lentiviral particles showed comparable levels of cell expression. Scale bar 400 µm. **(c)** SUM149 cells stably expressing Venus-p62^{DN} have a decrease of cell migration measured in Boyden chamber assays. Results are the mean ± S.D. of three experiments; Student's *t*-test, ***$P \leq 0.001$. **(d)** Pre-incubation of FITC-Tat-p62^{DN} peptide with SUM149 cell lysates inhibits formation of endogenous VANGL2-p62/SQSTM1 complex recovered with anti-VANGL2 antibody. A dose-dependent inhibition was evident between 5 and 25 µM of FITC-Tat-p62^{DN} but not scrambled peptide (FITC-Tat-Scr). **(e)** Assessment of cell proliferation following addition of FITC-Tat-p62^{DN} peptide or FITC-Tat-Ctrl peptide (225 µM each) to culture medium of breast cancer cells expressing high (SUM149 and HCC1806) and low (T47D) amount of VANGL2 by western blot. Equal amounts of p62/SQSTM1 are shown in these cells. Results are the mean ± S.D. of three experiments; Students *t*-test, *$P \leq 0.05$, ***$P \leq 0.001$, NS: not significant ($P > 0.05$).

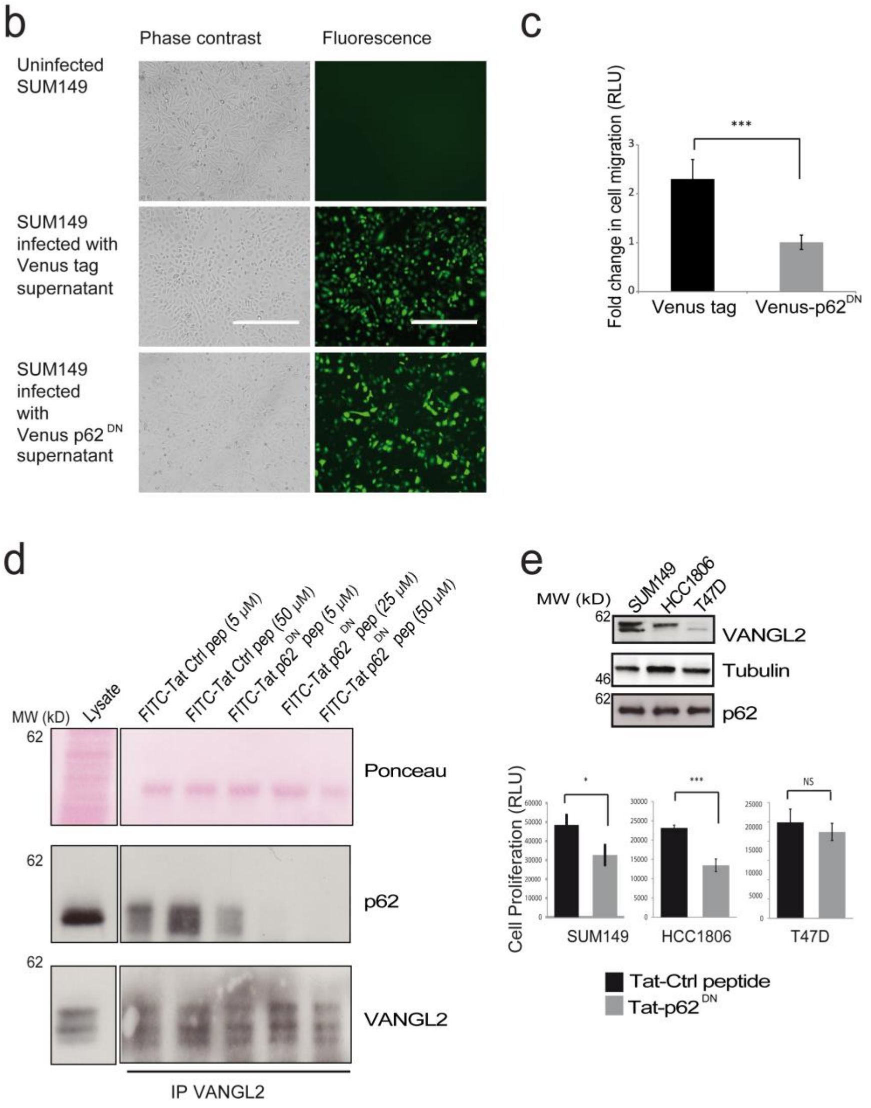

Supplementary Fig. 7 related to Fig. 7 (continued). The p62^{DN} peptide reduces cell migration, cell proliferation and JNK phosphorylation in breast cancer cells. (f) Proteins extracted from two PDX basal breast cancer samples expressing VANGL2 and p62/SQSTM1. VANGL2 was immunoprecipitated with anti-VANGL2 antibody and revealed with the indicated antibodies by Western blot. Equal quantities of tubulin loaded in the 2 lysates, as shown in full blot figure. (**g**) Protein extracts from PDX-derived cell lines under expressing ([VANGL2$_{low}$], PDX.DC 2 and 27) or overexpressing ([VANGL2$_{high}$] PDX.DC 13 and 26). VANGL2 were assessed by Western blot for expression of VANGL2, phosphorylated JNK, total JNK and tubulin. Quantification of pJNK/JNK ratio is provided (right panel). (**h**) PDX-derived cell lines were serum starved, pre-treated with Tat-JIP or Tat-p62^{DN} peptides, and stimulated with Wnt5a (100 ng ml^{-1}) for 5 min. No JNK phosphorylation was observed in serum-starved unstimulated cells. A decrease in JNK phosphorylation was noted in VANGL2 overexpressing PDX cell line (PDX.DC 26) treated with the two inhibitory peptides. Total JNK protein levels remain the same with and without inhibitors. Quantification of pJNK/JNK ratio is provided (right panel) for treatment of overexpressing PDX.DC 26.

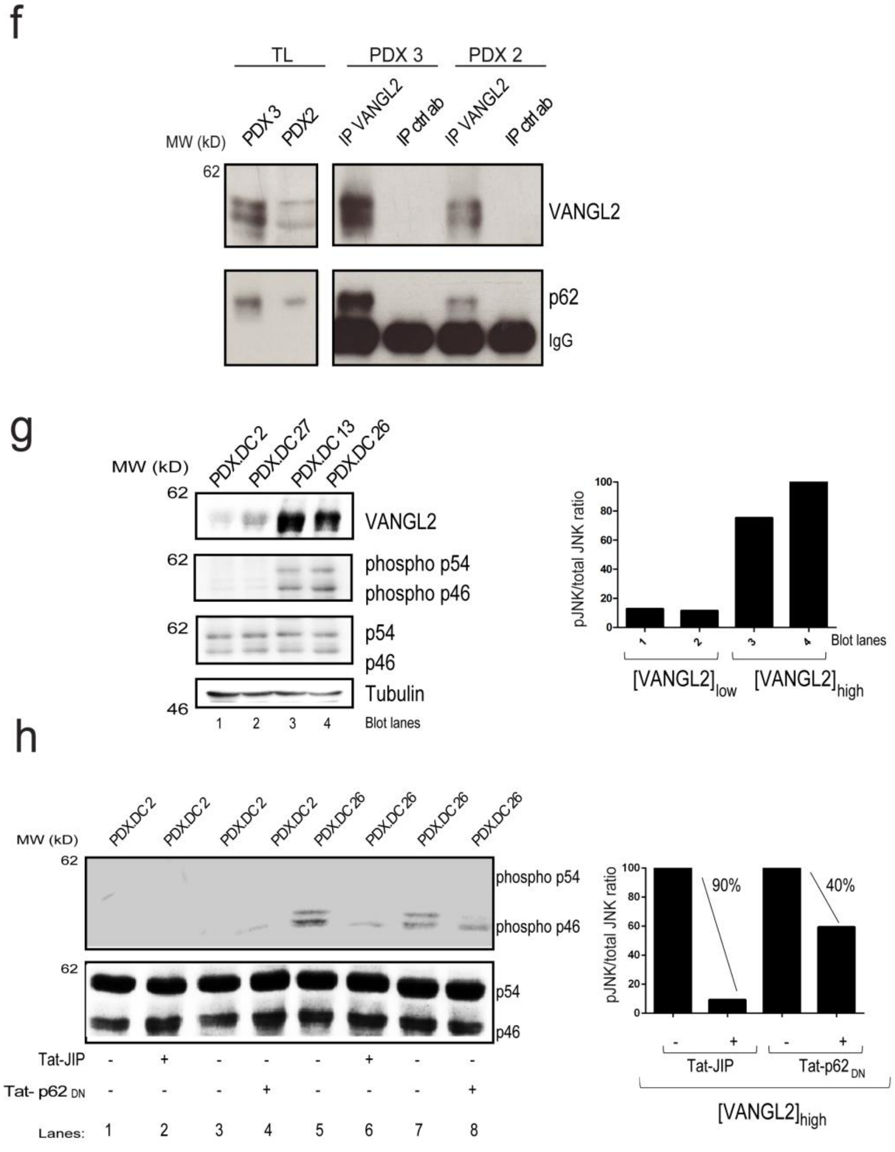

Supplementary Fig. 7 related to Fig. 7 (continued). Western blot analysis of patient-derived xenograft (PDX) breast tumours. (i) Western blot analysis of PDX breast tumours relating to **Fig. 7c and Fig. 7d**.

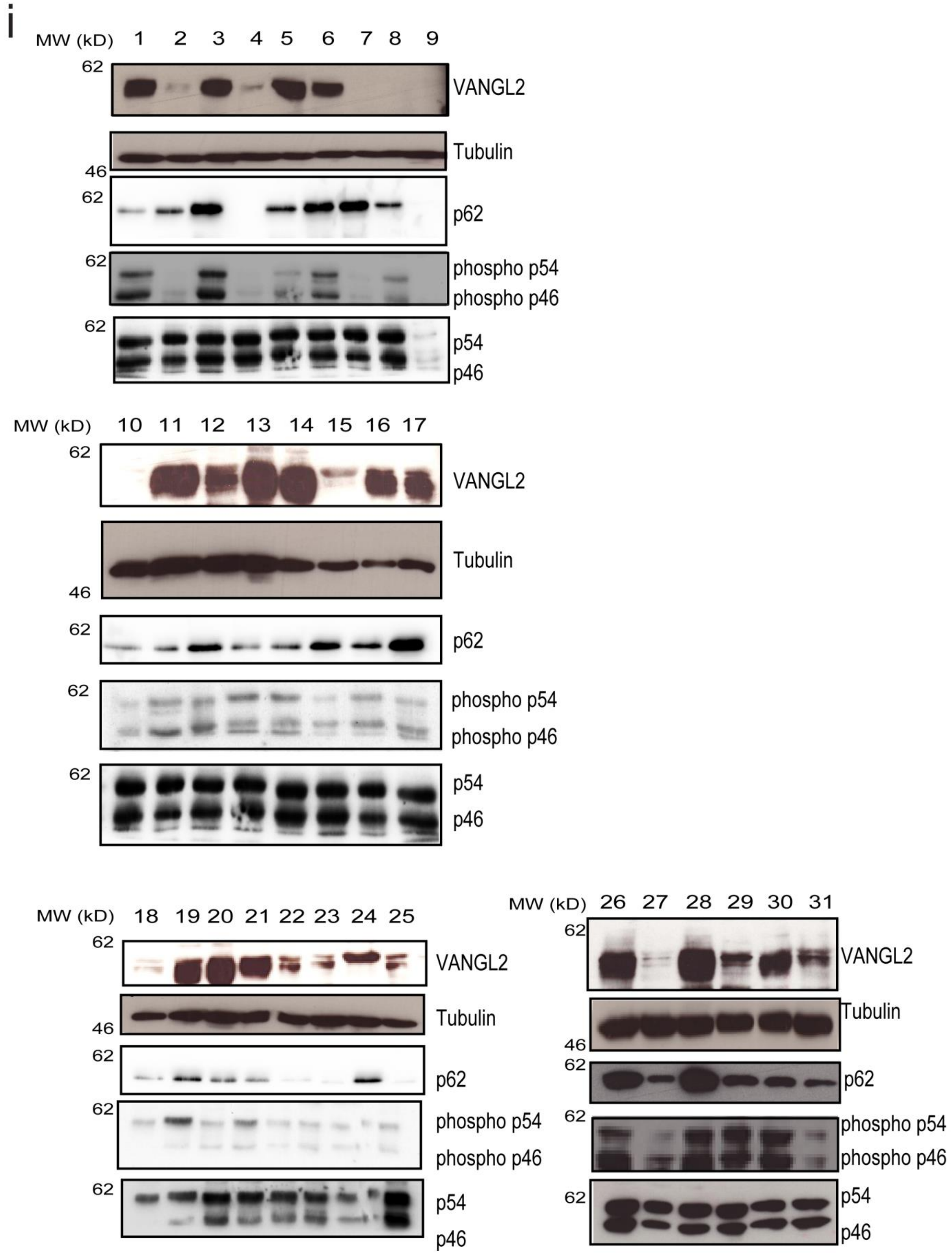

Full blots of main and supplementary figures. Full blots are provided for main and supplementary figures unless already shown in the figures themselves.

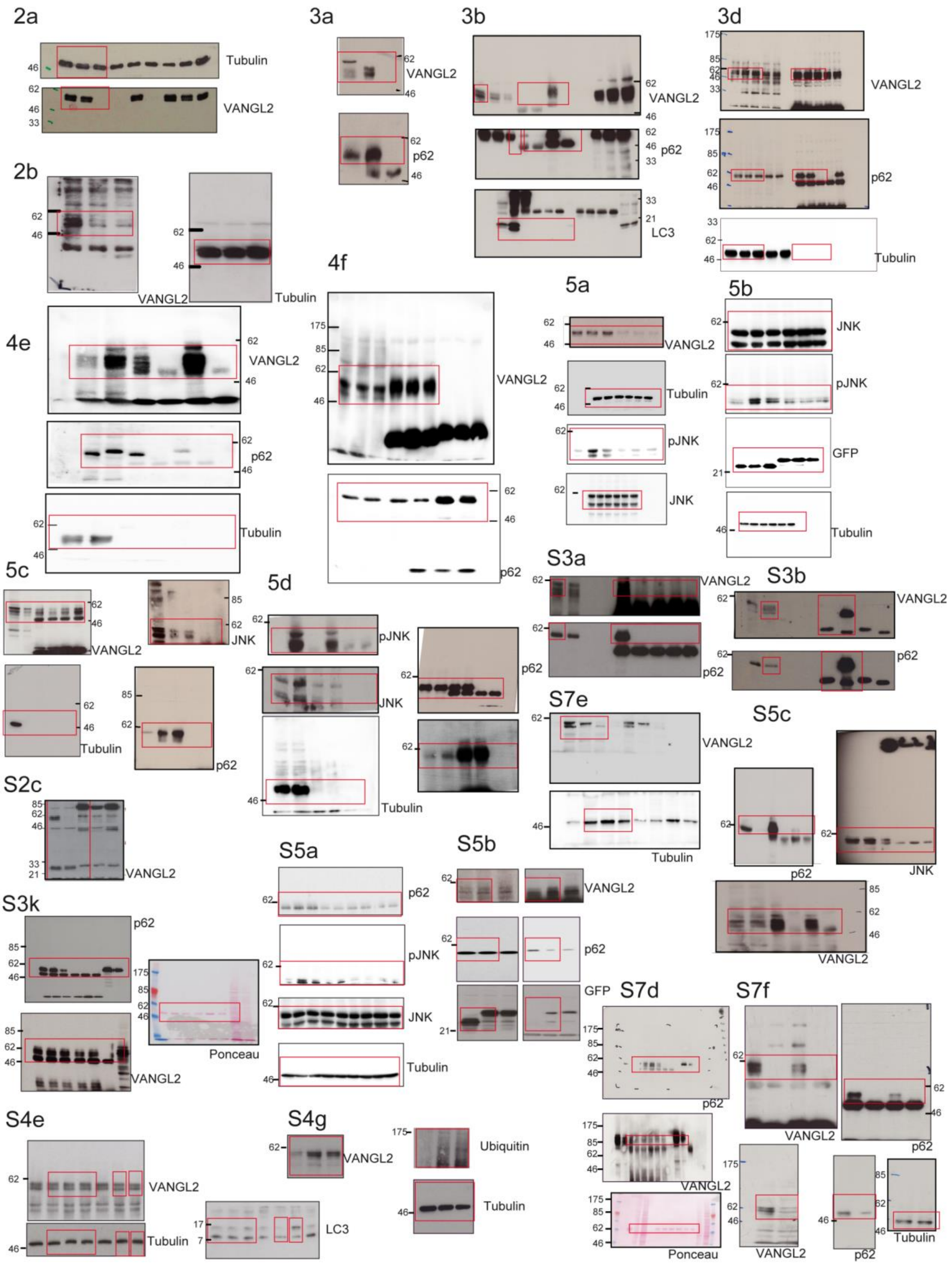

Supplementary Table 1: **Description of the breast cancer data sets used for gene expression analyses.**

Reference	Source of data	Samples* (N)	Technological platform	Probe sets (N)	*VANGL2* probe set ID**
van de Vijver et al., *NEJM* 2002	http://microarray-pubs.stanford.edu/wound_NKI/	254	Agilent Hu25K	25K	pos:148_117
van't Veer et al., *Nature* 2002	http://www.rii.com/publications/2002/vantveer.html	117	Agilent Hu25K	25K	pos:148_117
Expression Project for Oncology (expO) 2005	https://expo.intgen.org/geo GEO database GSE2109	348	Affymetrix U133 Plus 2.0	54K	226029_at
Ivshina et al., *Cancer Res* 2006	GEO database GSE4922	447	Affymetrix U133 A+B	2x22K	226029_at
Bonnefoi et al., *Lancet Oncol* 2007	GEO database GSE6861/GSE4779	125	Affymetrix X3P	61K	Hs.137507.0.S1_3p_at
Marty et al., *Breast Cancer Res* 2008	GEO database GSE13787	23	Affymetrix U133 Plus 2.0	54K	226029_at
Bos et al., *Nature* 2009	GEO database GSE12276	204	Affymetrix U133 Plus 2.0	54K	226029_at
Hoeflich et al., *Clin Cancer Res* 2009	GEO database GSE12763	30	Affymetrix U133 Plus 2.0	54K	226029_at
Barry et al., *J Clin Oncol* 2010	GEO database GSE23593	50	Affymetrix U133 Plus 2.0	54K	226029_at
Chen et al., *Breast Cancer Res Treat* 2010	GEO database GSE10780	42	Affymetrix U133 Plus 2.0	54K	226029_at
Korde et al., *Breast Cancer Res Treat* 2010	GEO database GSE18728	61	Affymetrix U133 Plus 2.0	54K	226029_at
Silver et al., *J Clin Oncol* 2010	GEO database GSE18864	84	Affymetrix U133 Plus 2.0	54K	226029_at
Desmedt et al., *J Clin Oncol* 2011	GEO database GSE16446	120	Affymetrix U133 Plus 2.0	54K	226029_at
Guedj et al., *Oncogene* 2011	Array Express database E-MTAB-365	452	Affymetrix U133 Plus 2.0	54K	226029_at
IPC series	GEO database GSE21653	330	Affymetrix U133 Plus 2.0	54K	226029_at

* only non redundant samples with *VANGL2* expression available were included in final analyses

** 100% specificity verified using the NCBI program BLASTN (2.2.27+)

Supplementary Table 2: *VANGL2* mRNA expression in breast cancer and histoclinical correlations.

Characteristics	N	*VANGL2* expression		p-value	Statistic
		no upregulation	upregulation		
Age (years)				0,379	0,91
≤50	956	721 (75%)	235 (25%)		[0.74-1.12]
>50	1118	862 (77%)	256 (23%)		
Pathological type				0,801	
ductal	737	530 (72%)	207 (28%)		
lobular	54	42 (78%)	12 (22%)		
mixed	19	13 (68%)	6 (32%)		
other	30	22 (73%)	8 (27%)		
Pathological tumor size (pT)				0,00985	1,4
pT1	519	409 (79%)	110 (21%)		[1.08-1.85]
pT2-3	833	604 (73%)	229 (27%)		
Pathological lymph node status (pN)				0,46	0,9
negative	570	409 (72%)	161 (28%)		[0.69-1.18]
positive	546	403 (74%)	143 (26%)		
Tumor grade				0,0671	1,3
1	314	257 (82%)	57 (18%)		[0.97-1.84]
2-3	1921	1483 (77%)	438 (23%)		
ER IHC status				1,76E-20	0,36
negative	663	426 (64%)	237 (36%)		[0.28-0.45]
positive	1246	1040 (83%)	206 (17%)		
PR IHC status				2,95E-09	0,49
negative	728	500 (69%)	228 (31%)		[0.39-0.63]
positive	829	677 (82%)	152 (18%)		
ERBB2 IHC status				0,0174	0,62
negative	829	619 (75%)	210 (25%)		[0.41-0.93]
positive	207	171 (83%)	36 (17%)		
Triple-negative (TN)				2,9E-13	3,3
no	1389	1152 (83%)	237 (17%)		[2.42-4.61]
yes	206	122 (59%)	84 (41%)		
Molecular subtype (PAM50)				0,0005	
luminal A	769	644 (84%)	125 (16%)		
basal	662	327 (49%)	335 (51%)		
ERBB2	418	368 (88%)	50 (12%)		
luminal B	515	451 (88%)	64 (12%)		
normal-like	323	272 (84%)	51 (16%)		
5-year MFS	1208	64% [0.61-0.67]	55% [0.05-0.62	0,00404	

Supplementary Table 3: Univariate and multivariate analyses for MFS.

		Univariate			Multivariate		
		N	HR [95CI]	*p* -value	N	HR [95CI]	*p* -value
**Age**	>50 vs ≤50 years	853	1.01 [0.78-1.3]	0.954			
**Pathological type**	lobular vs ductal	308	1.53 [0.78-2.97]	0.213			
	mixed vs ductal		0.59 [0.21-1.63]	0.308			
	other vs ductal		0.55 [0.17-1.76]	0.314			
**Pathological tumor size (pT)**	pT2-3 vs pT1	688	1.88 [1.4-2.5]	2.04E-05	325	0.98 [0.58-1.64]	0.934
**Pathological lymph node status (pN)**	positive vs negative	484	1.95 [1.42-2.66]	2.90E-05	325	2.28 [1.37-3.81]	1.59E-03
**Tumor grade**	2-3 vs 1	986	3.6 [2.37-5.47]	1.90E-09	325	3.61 [1.3-10.02]	1.38E-02
**ER IHC status**	positive vs negative	744	0.53 [0.41-0.69]	2.38E-06	325	0.58 [0.27-1.26]	0.170
**PR IHC status**	positive vs negative	392	0.58 [0.4-0.84]	3.33E-03	325	1.33 [0.62-2.84]	0.464
**ERBB2 IHC status**	positive vs negative	376	1.92 [1.29-2.86]	1.24E-03	325	1.06 [0.55-2.05]	0.860
***VANGL2* mRNA expression**	upregulation vs no upregulation	1208	1.34 [1.1-1.63]	4.11E-03	325	2.01 [1.22-3.29]	5.76E-03

N, number of samples with data available; HR, hazard ratio;95CI,95% confidence interval.

Supplementary References

1 Johansen, T. & Lamark, T. Selective autophagy mediated by autophagic adapter proteins. *Autophagy* **7**, 279-296, (2010).

2 Lamark, T. *et al.* Interaction codes within the family of mammalian Phox and Bem1p domain-containing proteins. *J Biol Chem* **278**, 34568-34581, (2003).
